# *Cinchona* alkaloid copolymers as fluorimetric INHIBIT and colorimetric AND logic gates for detection of iodide†

**DOI:** 10.1039/d5ra01281c

**Published:** 2025-04-08

**Authors:** Nicola' Agius, Catherine J. Ashton, Helen Willcock, David C. Magri

**Affiliations:** a Department of Chemistry, Faculty of Science, University of Malta Msida MSD 2080 Malta david.magri@um.edu.mt; b Department of Materials, Loughborough University Leicestershire LE11 3TU England UK

## Abstract

Four *cinchona* alkaloid-acrylamide water soluble copolymers with a mean hydrodynamic diameter of 3 nm were synthesised by free radical polymerization. The copolymers were characterised by ^1^H NMR, FTIR, GPC, DLS, UV-vis and fluorescence spectroscopy. A blue emission is observed with H^+^ switching of 185 and 175-fold for the quinidine and quinine copolymers, and 21 and 11-fold for the cinchonine and cinchonidine copolymers, while the presence of Cl^−^, Br^−^ or I^−^ causes fluorescence quenching. In emission mode, the copolymers function as fluorescent H^+^, X^−^-driven INHIBIT logic gates (where X = Cl^−^, Br^−^ or I^−^). In absorbance mode, the copolymers function as colorimetric H^+^, I^−^-driven AND logic gates in 1 : 1 (v/v) THF/water with a 76-fold enhancement. The solution colour changes from colourless to yellow with formation of new absorbance bands at 288 nm and 353 nm due to a π-anion non-covalent charge transfer interaction. The copolymers may be useful as selective iodide sensors for medical and analytical diagnostics.

## Abstract 2

*Cinchona* alkaloid-acrylamide copolymers are demonstrated as dual-sensing fluorescent INHIBIT and colorimetric AND logic gates in water.

## Graphical abstract

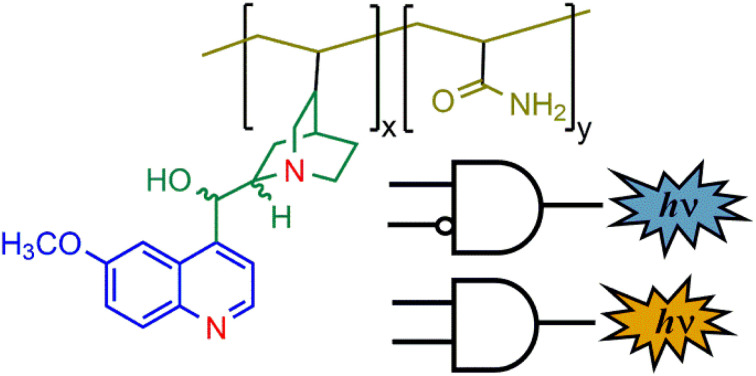

## Introduction

**Fig. 1:**
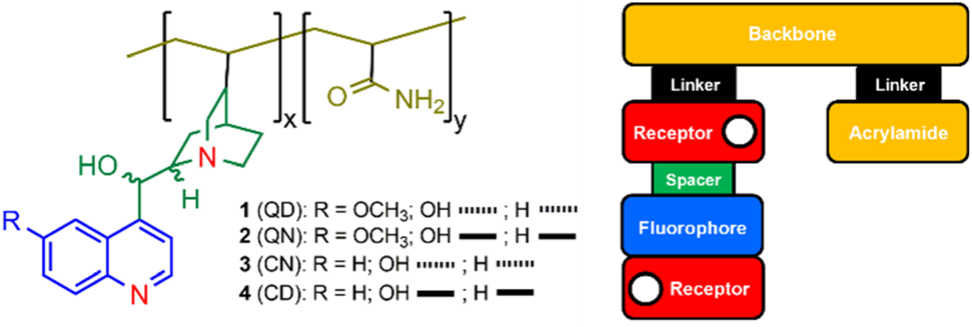
The *cinchona* alkaloid copolymers 1–4 (two-letter abbreviation in parentheses): QD = quinidine, QN = quinine, CN = cinchonine, CD = cinchonidine. The dashed and solid lines indicate the 3-dimensional stereochemistry of the OH and H groups. The colour-coded box diagram highlights the modular design within the polymeric logic gates. The copolymer ratios for *x* and *y* are given in Table 1.

Fluorescent natural products play a central role in many biological and medicinal processes, of which a mere 300 are intrinsically fluorescent.^1^ This exclusive group of compounds is extraordinary with oversized applications in theranostic nanomedicine, tissue imaging and photodynamic therapy.^2,3^ Another ambition is the development of optical probes derived from fluorescent natural products.^4–7^ Arguably, the oldest of these fluorescent natural products are the *cinchona* alkaloids, in particular the diastereomeric pair, quinine (QN) and quinidine (QD), which have medicinal uses as an antimalarial and antiarrhythmia, respectively.^8^ Quinine is also a prominent fluorescence quantum yield standard.^3^ Their less popular diastereomeric relatives, cinchonine (CN) and cinchonidine (CD), are near duplicates, except for the replacement of the methoxy group with a hydrogen atom (Fig. 1). The fascination with the *cinchona* alkaloids remains a topical subject due to their potential utility.^9–16^ For example, Reineke developed copolymers of quinine^13^ and hydroquinine^14^ for gene delivery and diagnostic imaging of polymer-pDNA binding. Yang reported a quinine-based quaternised copolymer as a drug-resistant antibacterial.^15^ Chen demonstrated *cinchona* alkaloid copolymers as circularly polarized organic afterglow smart materials for anticounterfeiting applications.^16^

An offshoot of fluorescent probes and luminescent sensors^17,18^ is the field of molecular logic-based computation.^19–25^ While small molecule entities with logic functions are common, polymeric logic gates are scarce.^26–33^ The first example by de Silva was a polymeric AND logic gate for pH and *T* (temperature) with *N*-alkylacrylamides.^28^ This study effectively expanded the field of molecular logic to polymers. Soon after, a structurally related *T*, H^+^-driven INHIBIT logic gate polymer was reported by Pasparakis with glucosyloxyethyl moieties,^29^ while an enzyme-driven lipase, β-galactosidase INHIBIT gate with a poly(phenyl) fluorene backbone was reported by Xing and Wang.^30^ Other examples include a poly(vinyl) alcohol Fe^3+^, F^−^-driven IMPLICATION (IMP) logic gate by Chowdhury,^31^ a methylmethacrylate F^−^, HSO_4_^−^-driven INHIBIT/IMP logic gate by Jiang,^32^ and a three-input *T*, H^+^, Cu^2+^-driven inverted enabled-OR gate by Tian.^33^ However, up to now, polymeric logic gates derived from fluorescent natural products are almost non-existent.^34^ Herein we demonstrate fluorescent natural product-based polymers as sustainable sourced intelligent materials.

In our earlier communication,^35^ we reported a fluorescent polymeric INHIBIT logic gate derived from quinidine 1 in water. We exploited the vinyl moiety to prepare an acrylamide copolymer by free radical polymerization.^36^ The copolymer was designed as a macromolecular logic gate with a *receptor*_1_*–fluorophore–spacer–receptor*_2_*–linker–backbone* blueprint (Fig. 1). The *cinchona* alkaloids remarkably incorporate an internal charge transfer (ICT) and photoinduced electron transfer (PET) mechanisms.^35,37^ Briefly, the modules within 1 are a 6-methoxyquinoline as the fluorophore, the quinoline nitrogen atom as receptor_1_, the hydroxylated ethane as the spacer, and the azabicyclic amine as receptor_2_. A satisfying outcome of our initial study was that the quinidine monomer and copolymer displayed identical photophysical properties, notably a bright fluorescent emission, indicating the optical functionality is conserved within the copolymer.^35^ We subsequently reported the logic-based fluorescent properties of the four *cinchona* alkaloids, quinidine, quinine, cinchonine and cinchonidine.^37^ In this study, we proceeded to study the logic properties of copolymers 1–4 (Fig. 1).

Herein we report the *cinchona* acrylamide copolymers 1–4 as dual sensing fluorimetric H^+^, X^−^-driven INHIBIT logic gates (where X = Cl^−^, Br^−^ or I^−^) and colorimetric H^+^, I^−^-driven AND logic gates. In emission mode, the copolymers operate in water with H^+^ as the enabling input, and Cl^−^ or another halide anion (Br^−^ or I^−^) as the disabling input. In absorbance mode, the copolymers cooperatively detect H^+^ and I^−^ in water, and optimally in 1 : 1 (v/v) THF/water, the copolymers provide a rapid, selective method for iodide detection.^38–47^ Being an essential dietary mineral, iodide is required for good health, notably for the prevention of thyroid diseases, such as goiter. Governmental health departments could find this technology useful for ensuring food and beverage producers adhere to the strict guidelines for iodide content in drinking water and food stuffs.

## Results & discussion

### Synthesis

**Scheme 1:**
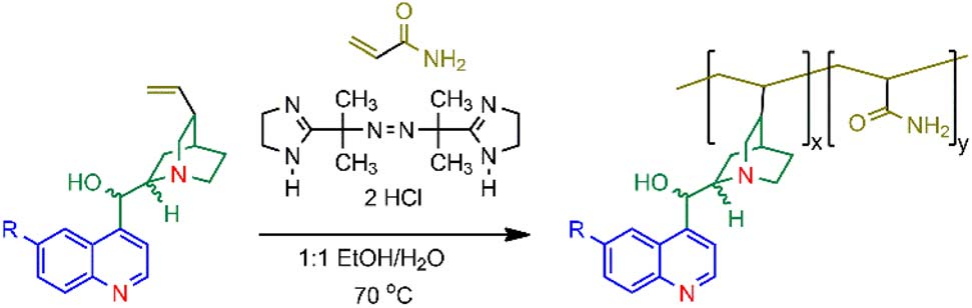
The synthesis of the *cinchona* alkaloid/acrylamide copolymers 1–4 using VA-044 (2,2′-azobis[2-(2-imidazolin-2-yl) propane]dihydrochloride) where R = OCH_3_ in 1 and 2 and R = H in 3 and 4. Refer to Fig. 1 for the stereochemistry.

The copolymers 1–4 were synthesised as shown in Scheme 1. A *cinchona* alkaloid and an excess of acrylamide were reacted in the presence of the radical initiator 2,2′-azobis[2-(2-imidazolin-2-yl)propane] dihydrochloride (VA-044) in 1 : 1 (v/v) H_2_O/EtOH. The synthesis of copolymer 1, initially prepared using ammonium persulfate as the free radical initiator,^35^ was repeated with VA-044 and found to provide a higher purity of the copolymer and improved synthetic reproducibility. Copolymers 1, 2 and 4 were reacted for 24 hours while 3 required 120 hours due to solubility issues. The copolymers were precipitated from solution with cold ethanol. The identity of the copolymers was accessed by ^1^H NMR and FTIR. Detailed characterisation data are available in the ESI (Fig. S1–S8†).

^1^H NMR was used to confirm the formation of the copolymers and to calculate the ratio of alkaloid to acrylamide units. The ^1^H NMR spectra of 1–4 in DMSO-*d*_6_ are noticeably broader than the *cinchona* monomers. While the proton chemical shifts and coupling constants are easily determined for the alkaloid monomers, such information was difficult to decipher for the copolymers as the peaks are broad due to the slower correlation time for rotational diffusion of polymers.^48^ The absence of the vinyl ABX pattern at 5.0–6.2 ppm in the ^1^H NMR spectra (Fig. S1–S4†) is evidence that the radical polymerisation reaction was successful. The FTIR spectra of the copolymers (Fig. S5–S8†) are noticeably broader than the monomers in the fingerprint region, and the appearance of an intense, broad peak at 1680 cm^−1^ is indicative of the primary amide C=O stretch from acrylamide in the polymer backbone.^49^ The composition ratios of the copolymers were estimated from the ^1^H NMR spectra by integrating the area of the quinoline proton at 8.70 ppm and the amide band at 6.80 ppm. The percentage of alkaloid is 17 ± 6% (acrylamide 82 ± 7%) in agreement with related quinine copolymers.^13^ We observe a stereochemical preference for a greater amount of acrylamide in the 1 and 3 copolymers. A comparison is provided in Table 1.

**Table 1:** Percent ratio of repeat units in copolymers 1–4^a^^,^^b^

	1 QD	2 QN	3 CN	4 CD
*Cinchona* alkaloid	14	23	11	21
Acrylamide (AM)	86	77	89	79

a Ratios determined by ^1^H NMR from integration of the quinoline H at 8.70 ppm and the broad NH_2_ singlet at 6.80 ppm in DMSO-*d*_6_.

b Quinidine (QD), quinine (QN), cinchonine (CN) and cinchonidine (CD).

### Gel permeation chromatography (GPC) and dynamic light scattering (DLS) analysis

The molecular number (*M*_n_), molecular weight (*M*_w_) and polydispersity index (*Đ*) were determined by GPC in water (Fig. S9 and Table S1†). Quinidine copolymer 1 has a *M*_n_ of 2400 Da with a *Đ* of 1.45. The *M*_n_ of 3 is similar at 2700 Da but has a larger *Đ* of 3.1 attributed to the longer reaction time. The diastereomeric copolymers 2 and 4 have a *M*_n_ of 1800 Da and 1900 Da, and a high *Đ* of 1.56 and 1.81. Using the ratios from the NMR data (Table 1) and the GPC data (Table S1†), the mean composition of the copolymers is three *cinchona* alkaloids and 18, 12, 27 and 15 AM repeat units for 1–4, respectively.

From dynamic light scattering (DLS),^50^ the hydrodynamic diameter of the copolymers is 3.0 nm in water. The polydispersity (PDI) of the diameters typically ranges between 0.186 and 0.539 indicating the copolymers are non-uniform in size (Table S2†). The correlation coefficients were good in all cases except for 3. To screen for the possibility of aggregate formation, concentration dependent studies were performed at pH 6 and pH 2. Samples of 5 mg mL^−1^ of copolymer 2 and 4 at pH 6 revealed the presence of particles with diameters of 87 nm and 58 nm, both with a PDI of 0.27. Dilution by 10-fold resulted in disassembly to 2 (PDI = 0.48), while particles remained for 4, but with narrower dispersity (PDI = 0.22). No assembly was observed at pH 6 with 1 and 3 at 5 mg mL^−1^. At pH 2 the copolymers showed no evidence of assembly at 5 mg mL^−1^, except in the case of 4, with particles of 72 nm, but on a two-fold dilution, they disassemble to 3.71 nm. At pH 6 the copolymers are monoprotonated at the quinuclidine N atom, while at pH 2 they are also protonated on the quinoline (*vide infra*).^37^ Hence, in acidic aqueous solution the copolymers are solvated by water molecules. These results are excellent considering that the photophysical studies were performed at concentration ≤0.12 g L^−1^ (Table 2). The DLS results clearly indicate that the UV-vis absorbance and fluorescence results (*vide infra*) are due to the intrinsic properties of copolymers 1–4 and not to self-assembled aggregates. DLS graphs are available in the ESI (Fig. S10–S14†).

**Table 2:** Photophysical properties of copolymers 1–4 in water^a^

	1 QD	2 QN	3 CN	4 CD
*λ*_Abs pH 11_/nm^b^	332	320	273	273
Log *a*_pH 11_^c^	2.86	0.89	3.49	1.17
*λ*_Abs pH 2_/nm^d^	352	345	316	315
Log *a*_pH 2_^c^	1.59	0.54	1.90	0.84
*λ*_Abs(isos)_/nm	261, 294, 329	263, 306	257, 304	305, 328
*λ*_Flu pH 11_/nm	389	382	381	400
*λ*_Flu pH 2_/nm	450	450	436	411
*λ*_Flu(isos)_/nm	393	390	381	349
p*K*_a_*^e^	4.08, 8.26	3.86, 8.20	3.80, 8.55	4.12^f^
FE^g^	185	175	21	11

a 0.078 g L^−1^1, 0.12 g L^−1^2, 0.063 g L^−1^3, 0.11 g L^−1^4.

b pH adjusted with 0.10 M TMAH.

c absorptivity units/cm^−1^ g^−1^ L.

d pH adjusted with 0.10 M CH_3_SO_3_H.

e Excited state p*K*_a_s determined by log[(*I*_max_ − *I*)/(*I* − *I*_min_)] = −log[H^+^] + log *K*_a_ from emission spectra in water buffered with 0.1 μM Na_2_EDTA. Fluorescence emission spectra obtained by excitation at *λ*_Isos_.

f Only one inflection point is observed from *I*–pH plot.

g H^+^-induced fluorescence enhancement (FE) *I*_FpH 2_/*I*_FpH 11_.

### Spectroscopic results

**Fig. 2:**
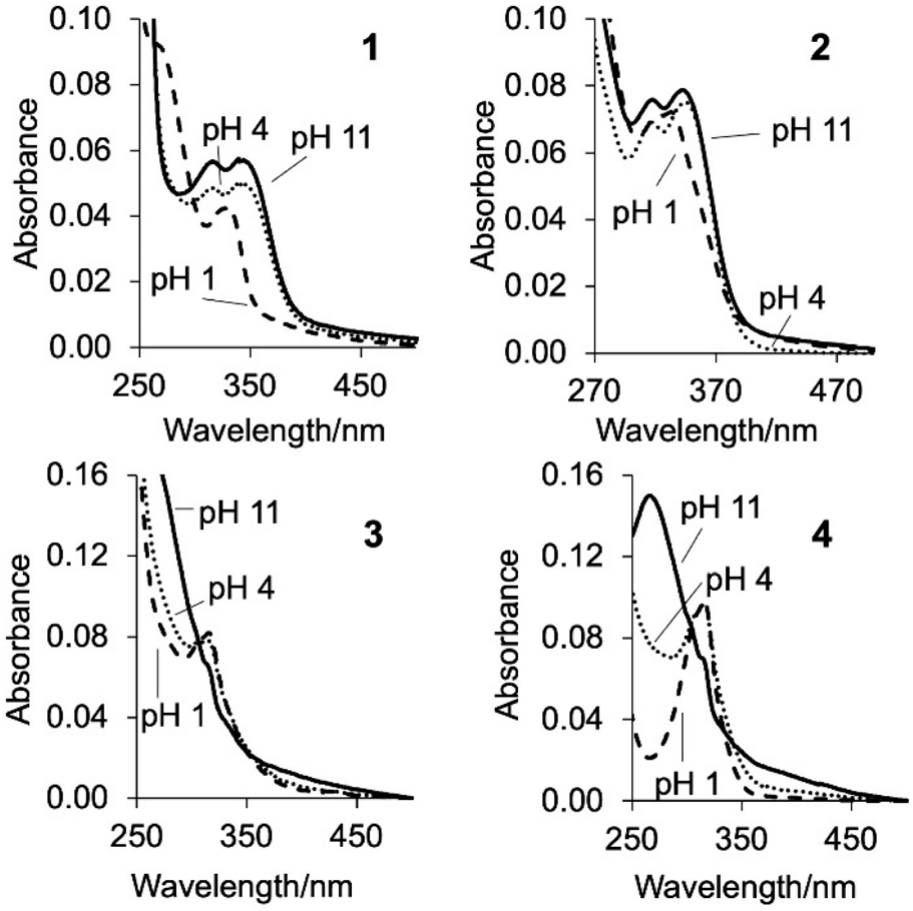
The UV-vis absorption spectra of copolymers 1–4 in water at pH 1, 4 and 11.

**Fig. 3:**
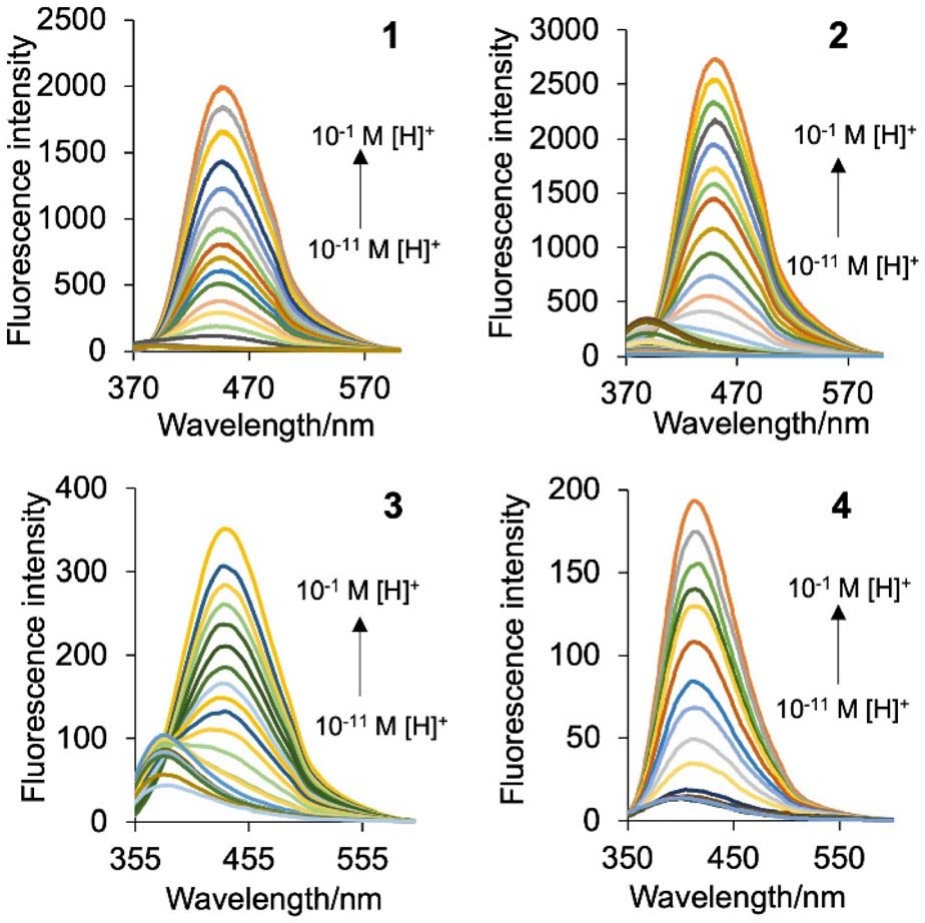
The emission spectra of copolymers 1–4 in water excited at 352 nm, 345 nm, 316 nm and 315 nm, respectively, with increasing H^+^.

The UV-visible absorption and emission properties of the copolymers 1–4 and the monomers^37^ were studied in water (Fig. 2, Table 2). Copolymers 1 and 2 have maximum absorbance bands (*λ*_Abs_) at 352 nm and 345 nm at 10^−2^ M H^+^, and 3 and 4 have peaks at 316 nm and 315 nm. These values are similar to the monomers confirming the acrylamide units do not contribute to the UV-vis spectrum. The monomers strongly absorb light with log *ε* (molar extinction coefficient) between 3.3-4.1 consistent with a π → π* electronic transition.^37^ The UV-vis properties of the copolymers were measured using absorptivity (*a*) in units of cm^−1^ g^−1^ L and determined to be 1.59, 0.54, 1.90 and 0.84 for 1–4, respectively. At 10^−11^ M H^+^, the log *a* increases to 2.86, 0.89, 3.49 and 1.17. A hypsochromic shift of *ca*. 30 nm reveals peak maxima at 332 nm, 320 nm, 273 nm and 273 nm. Isosbestic points are observed (listed in Table 2) consistent with protonation equilibria alluding to an ICT mechanism on protonation of the quinoline fragment. Fig. 3 highlights the emission spectra of copolymers 1–4. The spectra are broad ranging from 350–580 nm with a peak maximum at 450 nm, 450 nm, 436 nm and 411 nm for 1–4, respectively, in the presence of 10^−2^ M H^+^ consistent with monomer emission.^37^ At 10^−11^ M H^+^ the emission spectra of 1–4 are weak but accompanied by a bathochromic shift of 55 nm to 68 nm. As the titration proceeds, an isoemissive point appears at 393 nm, 390 nm, 381 nm and 349 nm. The fluorescence enhancement (FE) ratios from *I*_FpH 2_/*I*_FpH 11_ for 1 and 2 are 186 and 197, while those of 3 and 4 are an order of magnitude lower at 21 and 11. The greater enhancement for the methoxy *cinchona* derivatives suggests a stronger charge transfer character.

**Fig. 4:**
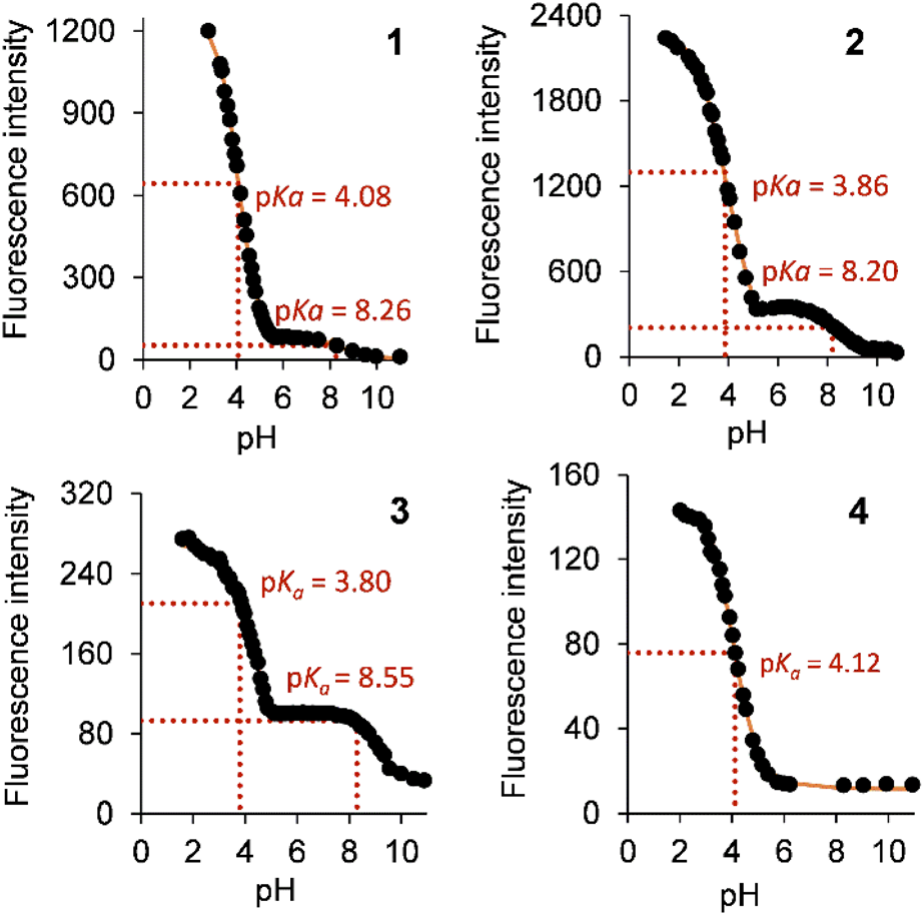
Maximum peak emission intensity–pH plots of copolymers 1–4 in water excited at 352 nm, 345 nm, 316 nm and 315 nm.

Fig. 4 illustrates the fluorescence intensity–pH plots for 1–4 based on the peak maximum. Copolymers 1–3 have a two-step sigmoidal curve whilst copolymer 4 has only a single-step sigmoidal curve. This same trend was observed with the monomers, due to the much lower fluorescence quantum yield (*Φ*_F_) of cinchonidine (*vide infra*).^37^ The excited state p*K*_a_s (p*K*_a_*s) of the quinoline and azabicyclic nitrogen atoms were evaluated to be 3.91 ± 0.17 and 8.35 ± 0.20 by fitting the data to the Henderson–Hasselbalch equation adapted for spectroscopic studies (Table 2, see footnote). These values are lower than the *cinchona* alkaloid monomers by 0.5–1.0 log unit. This observation suggests a less polar microenvironment about the alkaloids within the copolymer.

### Molecular logic by fluorescence

**Fig. 5:**
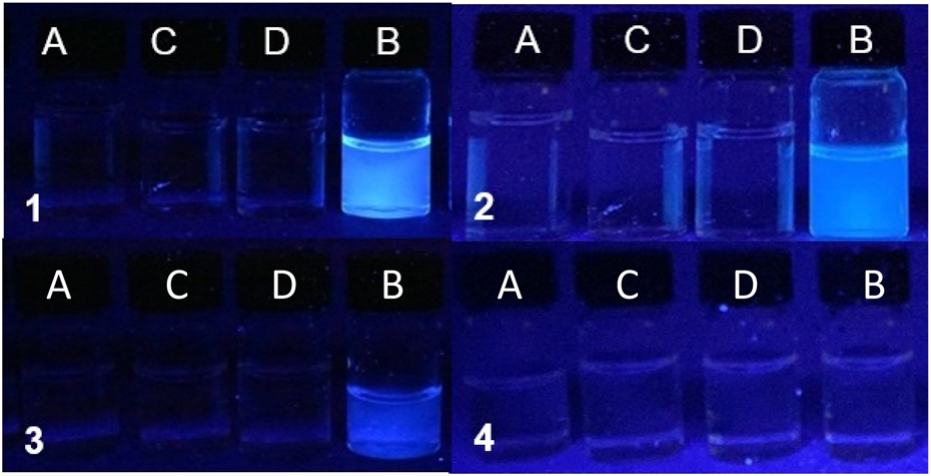
Acrylamide copolymers 1–4 irradiated with 365 nm UV light in water in the presence of 0.1 μM Na_2_EDTA. Condition (A) 10^−11^ M H^+^, (B) 10^−2^ M H^+^, (C) 10^−11^ M H^+^ & 100 mM Cl^−^ and (D) 10^−2^ M H^+^ & 100 mM Cl^−^.

**Fig. 6:**
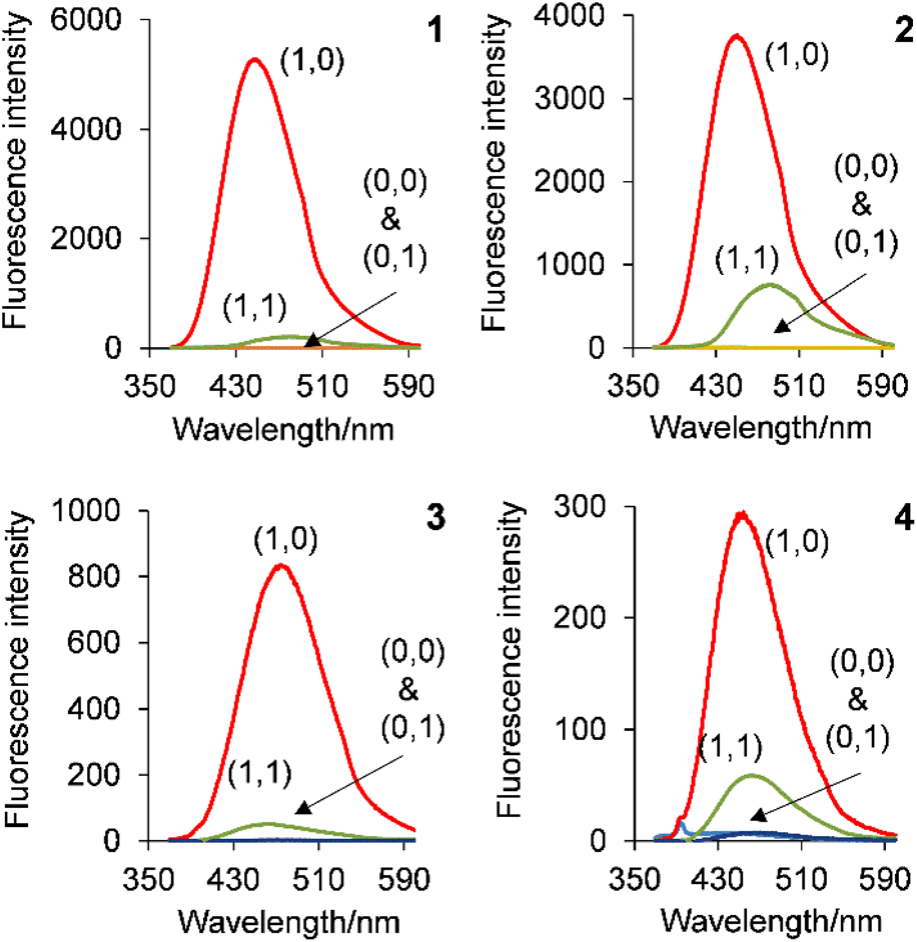
The emission spectra of 1–4 in water demonstrating H^+^, Cl^−^-driven INHIBIT logic.

Solutions of the copolymers 1–4 irradiated with 365 nm UV light are shown in Fig. 5 and the emission spectra are shown in Fig. 6. The inputs are H^+^ and Cl^−^ whilst the output is fluorescence. In all four cases, the copolymers function as H^+^, Cl^−^-driven INHIBIT logic gates. Initially at low H^+^ and Cl^−^, corresponding to the input state (0, 0), the emission is low due to PET from the azabicyclic amine. On addition of 10^−2^ M H^+^, the (1,0) input state, the emission significantly increases with FE ratios for 1 and 2 of 185 and 175-fold. The FE ratios of 3 and 4 are respectably good at 21 and 11-fold. The *Φ*_F_ of 1 and 2 are 0.553 and 0.549 resulting in a bright blue emission, while those of 3 and 4 are 0.046 and 0.025. In the absence of Cl^−^ (or even the presence of 1 mM Cl^−^), the (0,1) input state is an *off* state due to PET from the azabicyclic amine. The addition of 10^−2^ M H^+^ and 100 mM Cl^−^, the (1,1) input state, has a detrimental effect on the emission resulting in a similar output as the (0,0) input state. In all cases, the copolymers are non-fluorescent in the absence of H^+^ and Cl^−^, in the presence of Cl^−^, or in the presence of both H^+^ and Cl^−^. A high emission output is observed only when H^+^ is high. The truth tables for copolymers 1–4 are provided in Table 3.

**Table 3:** Truth tables for INHIBIT logic gates 1–4 with H^+^ and Cl^−^ in water^a^^,^^b^

Label	Input_1_ (H^+^)^c^	Input_2_ (Cl^−^)^d^	Output 1 (*Φ*_F_)^e^	Output 2 (*Φ*_F_)^e^	Output 3 (*Φ*_F_)^e^	Output 4 (*Φ*_F_)^e^
A	0 (low)	0 (low)	0 (0.003)	0 (0.004)	0 (0.004)	0 (0.001)
B	1 (high)	0 (low)	1 (0.553)	1 (0.549)	1 (0.046)	1 (0.025)
C	0 (low)	1 (high)	0 (0.003)	0 (0.003)	0 (0.004)	0 (0.002)
D	1 (high)	1 (high)	0 (0.003)	0 (0.006)	0 (0.004)	0 (0.004)

a 0.078 g L^−1^1, 0.12 g L^−1^ μM 2, 0.063 g L^−1^3, 0.11 g L^−1^4.

b Excited at 352 nm, 343 nm 315 nm, and 312 nm.

c High input_1_ 10^−2^ M H^+^ and low input_1_ H^+^ 10^−11^ M adjusted with CH_3_SO_3_H and TMAH.

d High input_2_ 100 mM Cl^−^ and low input_2_ 1 mM Cl^−^ added as NaCl.

e Relative *Φ*_F_*versus* 10^−6^ M quinine sulfate in aerated 0.1 M H_2_SO (*Φ*_F_ = 0.55). High threshold output level set at *Φ*_F max_/2.

Besides Cl^−^, Br^−^ or I^−^ anions also act as disabling inputs. The presence of 100 mM chloride, bromide or iodide anions, whether in acidic or basic media, causes a low emission output. Hence, all three halide anions disable input_1_ (H^+^). Therefore, the copolymers 1–4 can also be regarded as a three-input disabled OR logic gate feeding into the disabling input of INHIBIT logic gate. The logic behavior of the polymers remained identical to those of the monomer *cinchona* alkaloids. Therefore, modularity is conserved upon polymerisation with the acrylamide unit. The disabling ability of Cl^−^, Br^−^ or I^−^ anions has historically been explained by collisional quenching of the singlet excited state.^51^ The disabling power of the halides is according to the heavy atom effect whereby the order is I^−^ > Br^−^ > Cl^−^. However, an alternative rationale for the quenching process may be due to an intermolecular charge transfer (*vide infra*).

### Molecular logic by absorbance

A serendipitous finding during our study was that the copolymers slowly undergo a colour change in water in the presence of I^−^ at 10^−2^ M H^+^. After 48 hours the aqueous solutions of 1–4 changed from colourless to a faint yellow in the presence of 200 mM iodide (Fig. S17†). Inspection by UV-vis absorbance spectrometry revealed significant spectral changes as a function of time with new peaks at 288 nm and 353 nm (Fig. S15†). The monomer *cinchona* alkaloids under identical conditions also resulted in similar colour changes, although higher concentrations of I^−^ were needed. A review^47^ of the literature reveals precedence for this phenomenon with nitrogen-rich chemosensors containing pyridine,^38^ tripodal benzimidazole,^39^ acridine,^40^ fluorene-dipyridine,^41^*o*-phenylenediamine^42^ and fluorenone-imine-catechol.^43^ Copolymers of 1,5-naph thyridine,^44^ carbazole^45^ and phenylene vinylenes^46^ are reported as both colorimetric and fluorimetric I^−^ sensors. A commonality in these literature sources is that iodide sensing is typically demonstrated in aqueous THF solutions.^38–47^ Thus, we proceeded to test the response of copolymers 1–4 in THF/water compositions.

**Fig. 7:**
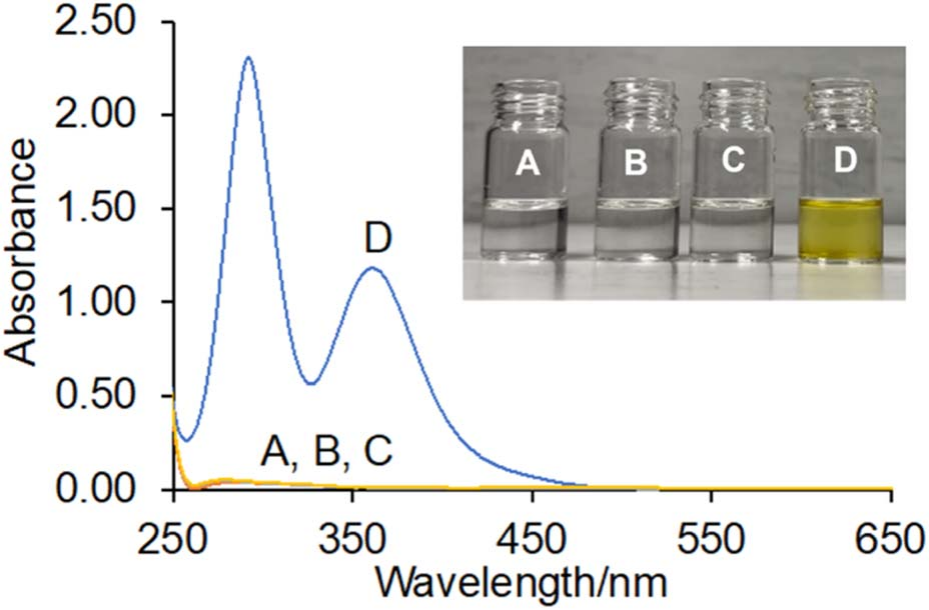
UV-vis absorbance spectra (solvent subtracted) of 0.12 g L^−1^ of poly(QN-*co*-AM) 2 in 1 : 1 (v/v) THF/water: (A) 10^−9^ M H^+^ (B) 10^−2^ M H^+^ (C) 10^−9^ M H^+^, 1 mM I^−^ (D) 10^−2^ M H^+^, 1 mM I^−^ after 10 min. The copolymer operates as a H^+^, I^−^-driven AND logic gate.

Copolymers 1–4 display colorimetric logic characteristics dependent on the solvent conditions. In 1 : 1 (v/v) THF/water, a yellow colour was observed with the naked eye only in the presence of 10^−2^ M H^+^ and 1 mM I^−^ after 10 min (Fig. 7 inset and Fig. S16†). No colour change was observed with 10^−9^ M H^+^ and 1 mM I^−^, or in the presence of only 10^−9^ M H^+^, or only 1 mM I^−^. Satisfyingly, these results in water (Fig. S19†) and 1 : 1 (v/v) THF/water (Fig. 7) are consistent with AND logic.^52^ The AND truth table with absorbance outputs are given in Table 4. Remarkably, the absorbance increases 76-fold.

**Table 4:** Truth table for copolymer logic gate 2 with H^+^ and I^−^ inputs and an absorbance (abs) output monitored at 360 nm^a^

Label	Input_1_ (H^+^)^b^	Input_2_ (I^−^)^c^	Output abs (1 : 1 THF/H_2_O)^d^	Output abs (9 : 1 THF/H_2_O)^d^
A	0 (low)	0 (low)	0 (low, 0.010)	0 (low, 0.013)
B	1 (high)	0 (low)	0 (low, 0.011)	0 (low, 0.010)
C	0 (low)	1 (high)	0 (low, 0.014)	1 (high, 1.05)
D	1 (high)	1 (high)	1 (high, 1.06)	1 (high, 3.94)

a 0.12 g L^−1^ μM 2.

b High and low input_1_ is 10^−2^ M and 10^−9^ M H^+^ adjusted with CH_3_SO_3_H and TMAH.

c High input_2_ in 1 : 1 THF/H_2_O is 1 mM I^−^. High input_2_ in 9 : 1 THF/H_2_O is 10 μM. I^−^ added as KI.

d High threshold output level set at abs >0.6.

By comparison, in 9 : 1 (v/v) THF/water, a yellow colour change was observed with 10 μM I^−^ after 1 min. With 10 μM I^−^ and 10^−2^ M H^+^ the solution colour is even darker (Fig. S18†). The resulting UV-vis spectra is so absorbent on addition of both inputs that the detector becomes saturated (Fig. S20,† and Table 4). In THF the colour change on addition of I^−^ is immediate. From a Boolean perspective, the copolymers now function as H^+^, I^−^-driven TRANSFER logic gates where I^−^ is the enabling input.^53^ Tuning of the solvent polarity, reconfigures the copolymers 1–4 from rapid colorimetric H^+^, I^−^-driven AND logic gates in 1 : 1 (v/v) THF/water to H^+^, I^−^-driven TRANSFER logic gates in 9 : 1 (v/v) THF/water.^54,55^ We also tested the response of the copolymers to F^−^ and observed no change in either the absorbance or emission output. Thus, the copolymers are selective colorimetric indicators for differentiating I^−^ from F^−^, Cl^−^ and Br^−^.

These observations are rationalized by an intermolecular charge transfer mechanism^56^ – more specifically, to a π-anion non-covalent interaction.^57,58^ Iodide is an electron-rich halide, more so than F^−^, Cl^−^ and Br^−^, which hold onto their electron density tighter. Hence, I^−^ ions are more able to share electron density with electron-deficient π-systems, such as the positively charged quinolinium units, of the *cinchona* alkaloids. The supramolecular interaction is faciliated by the electrostatic attraction between I^−^ and the positively charged π-system, and by an anion-induced polarization of the π-system.^59^ Furthermore, the cationic polymer environment amplifies an electric field effect due to the multiple quinoliniums units such that the iodide counter ions cause the copolymers to condense into a compact structure (similar to cation condensation with DNA).^60^ This explains the higher sensitivity (lower I^−^ concentrations) detected by the copolymers compared to the monomers.

## Conclusions

Four copolymers of *cinchona* alkaloids and acrylamide with a mean hydrodynamic diameter of 3 nm were synthesised by free radical polymerization. The copolymers are illustrative examples of H^+^, X^−^-driven INHIBIT fluorescent logic gates (where X = Cl^−^, Br^−^ or I^−^) in aqueous solution. A blue fluorescence is observed in the presence of high H^+^, while the presence of Cl^−^, Br^−^ or I^−^ disables the emission, irrespective of whether H^+^ is present. The QD and QN copolymers exhibit fluorescence quantum yields (*Φ*_F_ = 0.55) equivalent to the monomers,^3,37^ which are more intense than the CN (*Φ*_F_ = 0.046) and CD (*Φ*_F_ = 0.025) copolymers. The assimilation of the *cinchona* alkaloids into the acrylamide backbone does not interfere with the intrinsic fluorescence of the alkaloids. Hence, the optical properties of the *cinchona* alkaloids are conserved within the copolymer environment. Additionally, the copolymers function as rapid and selective colorimetric H^+^, I^−^-driven AND logic gates in absorbance mode in aqueous THF solution. Iodide is detected with the naked eye by a solution colour change from colourless to yellow due to a π-anion non-covalent interaction between iodide and the quinolinium fragment.^56,57^ Studies are currently underway to better understand this supramolecular interaction.

## Author contributions

Conceptualization (DCM), investigation (NA, CJA), methodology (DCM, HW), formal analysis (NA, CJA, DCM), supervision (DCM, HW), writing – original draft (DCM, NA), writing – review & editing (NA, CJA, HW, DCM).

## Conflicts of interest

There are no conflicts to declare.

## Supplementary Material

RA-015-D5RA01281C-s001
