# Supplementary material for: Cinchona alkaloid copolymers as fluorimetric INHIBIT and colorimetric AND logic gates for detection of iodide
Source: RSC Adv. 2025 Apr 8;15(14):11121–7. doi: 10.1039/d5ra01281c (PMC11977102; doi:10.1039/d5ra01281c)
Supplement: RA-015-D5RA01281C-s001 [file RA-015-D5RA01281C-s001.pdf]

Supplementary Information (SI) for RSC Advances.
This journal is © The Royal Society of Chemistry 2025

# Supporting Information

## *Cinchona* Alkaloid Copolymers as Fluorimetric INHIBIT and Colorimetric AND Logic Gates for Detection of Iodide

Nicola' Agius,[a] Catherine J. Ashton,[b] Helen Willcock[b] and David C. Magri[a]*

[a] Department of Chemistry, Faculty of Science, University of Malta, Msida, MSD 2080, Malta.

[b] Department of Materials, Loughborough University, Leicestershire, LE11 3TU, England, UK.

*Corresponding author, email Address: david.magri@um.edu.mt

# Table of Contents

**Chemicals**……………………………………………………………………………… 2

**Instrumentation**……………………………………………………………………….... 2

**Synthesis & Characterisation**…………………………………………………………… 3

**Fig. S1** $^1$H NMR spectrum of **1** in DMSO-$d_6$………………………………………………… 5

**Fig. S2** $^1$H NMR spectrum of **2** in DMSO-$d_6$………………………………………………… 6

**Fig. S3** $^1$H NMR spectrum of **3** in DMSO-$d_6$………………………………………………… 7

**Fig. S4** $^1$H NMR spectrum of **4** in DMSO-$d_6$………………………………………………… 8

**Fig. S5** IR spectrum of **1** (KBr disc)……………………………………………………………9

**Fig. S6** IR spectrum of **2** (KBr disc)……………………………………………………………10

**Fig. S7** IR spectrum of **3** (KBr disc)…………………………………………………………… 11

**Fig. S8** IR spectrum of **4** (KBr disc)…………………………………………………………… 12

**Fig. S9** Gel permeation chromatography of copolymers **1-4** in water………………………..13

Table **S1**. The molecular weights and polydispersity indexes for copolymer **1-4**………..…13

**Fig. S10** DLS of copolymer **1** QD-co-AM as a function of concentration and pH………..…..14

**Fig. S11** DLS of copolymer **2** QN-co-AM as a function of concentration at pH 6……..……15

**Fig. S12** DLS of copolymer **2** QN-co-AM as a function of concentration at pH 2…..………16

**Fig. S13** DLS of copolymer **3** CN-co-AM as a function of concentration and pH……..……17

**Fig. S14** DLS of copolymer **4** CD-co-AM as a function of concentration and pH……….....18

**Fig. S15** UV-vis absorbance spectra of **2** in water……………..…………………………19

**Fig. S16** UV-vis absorbance spectra of **2** in 1:1 (v/v) THF/$H_2O$……..………………………19

**Fig. S17** Vials of **2** demonstrating AND logic function in water……..…………………….....20

**Fig. S18** Vials of **2** demonstrating TRANSFER logic in 9:1 (v/v) THF/water……………....20

**Fig. S19** UV-vis spectra of **2** demonstrating AND logic in water…………………..……..21

**Fig. S20** UV-vis spectra of **2** demonstrating TRANSFER logic in 9:1 (v/v) THF/water…….21

**Table S2**. The polydispersity index values for copolymers **1-4** ..........................................22

## Experimental

### Chemicals

Acetone (GPR Grade, LEVO), acetonitrile (HPLC Grade, Fischers), acrylamide (99%, Merck), ammonium persulfate (99%, Sigma-Aldrich), 2,2'-azobis[2-(2-imidazolin-2-yl)propane]dihydrochloride (98%, TCI), cinchonine (98%, TCI), cinchonidine (98%, TCI), celite (HPLC, Across Organics), DMSO-$d_6$ (99.8%, VWR), diethyl ether (HPLC, Carlo Erba), dichloromethane (HPLC, Carlo Erba), Disodium ethylenediaminetetraacetic acid ($Na_2EDTA$), ethyl acetate (HPLC, Carlo Erba), ethanol (HPLC, Carlo Erba), EDTA (99%, Pancreac), hydrochloric acid (37%, Fischer Scientific), methanesulfonic acid (99.5%, TCI), pentane (HPLC, Carlo Erba), potassium bromide (99.8%, Sigma-Aldrich), potassium permanganate (99%, Fluka), potassium persulfate (GPR, BDH), potassium iodide (99 %, VWR), quinine (98 %, TCI), quinidine sulfate dihydrate (98 %, TCI), sodium chloride (98%, Scharlau), sodium hydroxide (98%, Fluka), triethylamine (Synthesis, Scharlau), tetrahydrofuran (HPLC, Carlo Erba), tetramethylammonium hydroxide (TMAH, 98% in water, Fluka). 4 Å molecular sieves (Thermo scientific) activated in a furnace at 350 °C for 24 hours before use. Silica gel 60A (70-230 mesh, Carlo Erba) and silica on TLC aluminium foil (fluorescent indicator 254 nm, Sigma-Aldrich) were used for column and thin-layer chromatography.

### Instrumentation

Reactions were carried out in either a one-neck or two-neck round bottom flasks (50 mL, 100 mL or 250 mL) partially immersed in mineral oil and heated using an IKA C-MAG HS 7 hotplate with IKA ETS-D5 temperature probe. Rotary evaporator connected to a Laboport N 815 mini diaphragm vacuum pump and a Stuart RE300DB digital water bath. An international Clinical Centrifuge Model 40652H was used to separate the product from the solvent for the work-up of the acrylamide copolymers. $^1$H NMR spectra were recorded in $CDCl_3$ or DMSO-$d_6$. A Bruker Avance III HD 500 MHz NMR spectrometer equipped with an Ascend 500 11.75 Tesla Superconducting magnet at a frequency of 500.13 MHz for $^1$H NMR with a multinuclear 5 mm PABBO probe. Jasco V-650 spectrophotometer and 1.0 cm pathlength quartz cells were used to obtain UV-vis absorption spectra. Parameters were set at 1.0 nm bandwidth and scan speed at 400 nm/min. Samples were scanned over the range of 250-500 nm. A blank background run was performed for all spectra to correct for solvent. Fluorescence emission spectra were performed using a Jasco FP-9300 spectrophotometer. Naked-eye fluorescence was observed using a UVGL-58 handheld lamp with long wavelength

(365 nm) and short wavelength (254 nm) functions. The pH of the solutions used for UV and fluorescence titrations were recorded by Hanna instrument 210 microprocessor pH meter after calibrating with buffer solutions at pH 4.00 and 7.00. Prior to obtaining fluorescence spectra, a blank run was performed using air and water to correct for the solvent. A 1.0 cm pathlength (QS) quartz cuvette with optically clear sides was used. Excitation and emission bandwidths were set at 2.5 nm, response time of 50 msec, and scanning speed 200 nm/min. Size-exclusion chromatography (SEC, GPC) was done on a PL-aquag-OH PL1110-6504 column at a flow rate of 1.0ml/min at 30 °C in water/0.1 M $NaNO_3$ using polyethylene glycol (PEG) as the standard. DLS measurements were performed using a Malvern Zetasizer Nano NS and Zetasizer software, version 7.12. The system uses an avalanche photodiode detector and a diode-pumped solid-state laser operating (wavelength of 532 nm). The light scattered was detected at a 175° angle, and the temperature was stabilised at 25 ± 0.1 °C. Particle suspensions were diluted to 10 μL in 1 mL deionised water and filtered through 400 nm nylon syringe filters.

**Synthesis and Characterisation**

Reactions were performed in two-necked 100 mL round-bottom flask in 5 mL of 1:1 (v/v) $H_2O$/EtOH fitted with a water condenser and purged with nitrogen gas with a stainless needle for 5 min. The reaction was maintained under a positive nitrogen atmosphere as indicated by a balloon fitted to the top of a condenser.

**Synthesis of 1, Poly(QD-co-am)**

Quinidine sulfate dihydrate (0.30 g, 0.38 mmol), acrylamide (0.10 g, 1.4 mmol) and VA-044 (0.020 g, 0.062 mmol) were dissolved in 5 mL of 1:1 (v/v) $H_2O$/EtOH. The reaction was stirred at 70 °C for 24 hours. Addition of cold EtOH to the brown coloured resulted in the precipitation of a white powder. After centrifuging, ethyl acetate was added to aid the removal of EtOH by rotary evaporator to give an off-white solid (30 mg). $R_f$ = 0.11 (9:1 (*v/v*) $CH_2Cl_2$/MeOH); $^1$H NMR (DMSO-$d_6$, ppm): δ 8.78-8.66 (1H), 7.98-7.88 (1H), 7.73-7.02 (2H), 7.02-6.63 (2H), 5.16 (1H), 4.00-3.92 (4H), 2.35-1.16 (12H); IR (KBr, $cm^{-1}$): 3400-2000, 1681-1504, 1477-1000.

**Synthesis of 2, Poly(QN-co-Am)**

Quinine (0.30 g, 0.92 mmol), acrylamide (0.10 g, 1.4 mmol) and VA-044 (0.020 g, 0.062 mmol) were dissolved in 5 mL 1:1 $H_2O$/EtOH. The reaction was stirred at 70 °C for 24 hours. The work-up was as described for **1**. Poly(QN-co-Am). **2** was collected as

an off-white powder (60 mg). $R_f$= 0.11 (9:1 (v/v) $CH_2Cl_2$/MeOH); $^1H$ NMR (DMSO-$d_6$, ppm): $\delta$ 8.72 (1H), 7.95 (1H), 7.62-7.00 (3H), 7.00-6.65 (2H), 4.98 (1H), 4.36 (1H), 3.98-4.00 (3H), 2.57-1.21 (12H); IR (KBr, $cm^{-1}$): 3600-3080, 1681-1504, 1477-1000.

**Synthesis of 3, Poly(CN-co-Am)**

Cinchonine (0.30 g, 1.0 mmol), acrylamide (0.10 g, 1.4 mmol) and VA-044 (0.020 g, 0.062 mmol) were dissolved in 5 mL 1:1 $H_2O$/EtOH. The reaction was stirred at 70 °C for 5 days (due to poorer solubility). The work-up was as described for **1**. Poly(CN-co-Am). **3** was collected as a yellowish powder (20 mg). $R_f$ = 0.15 (9:1 (v/v) $CH_2Cl_2$/MeOH); $^1H$ NMR (DMSO-$d_6$, ppm): $\delta$ 8.96-8.87 (d, 1H, $J$ = 4.45 Hz), 8.38-8.30 (d, 1H, $J$ = 8.55Hz), 8.12-8.06 (d, 1H, $J$ = 8.10 Hz), 7.83-7.76 (t, 1H, 7.33), 7.72-7.65 (t, 1H, 7.70), 7.48-6.67 (2H), 5.16 (1H), 3.88 (1H), 2.41-1.23 (12H); IR (KBr, $cm^{-1}$): 3600-3080, 1681-1500, 1477-1100.

**Synthesis of 4, Poly(CD-co-Am)**

Cinchonidine (0.30 g, 1.0 mmol), acrylamide (0.10 g, 1.4 mmol) and VA-044 (0.020 g, 0.062 mmol) were dissolved in 5 mL 1:1 $H_2O$/EtOH. The reaction was stirred at 70 °C for 24 hours. The work-up was as described for **1**. Poly(CD-co-Am). **4** was collected as an off-white powder (70 mg). $R_f$ = 0.14 (9:1 (v/v) $CH_2Cl_2$/MeOH); $^1H$ NMR (DMSO-$d_6$, ppm): $\delta$ 9.06-8.91 (1H), 8.48-8.40 (1H), 8.16-8.06 (1H), 7.89-7.63 (3H), 7.55-6.69 (2H), 5.06 (1H), 4.42 (1H), 2.49-1.16 (12H); IR (KBr, $cm^{-1}$): 3750-3080, 1681-1504, 1477-1000.

**Spectroscopic Experiments**

Solutions of copolymer **1-4** were prepared in 1 M methanesulfonic acid at an absorbance of 0.1. The concentrations were 0.078 g/L of **1**, 0.12 g/L of **2**, 0.063 g/L of **3** and 0.11 g/L of **4**. Each solution contained $5\times10^{-9}$ M EDTA as a buffer and to chelate to trace metal ions. The solution pH was monitored with a calibrated pH meter using buffer solutions at pH 4 and pH 7. The basicity was adjusted using either 1 M, 100 mM, 10 mM, 1 mM or 0.1 mM $(CH_3)_4NOH$. Solutions were stirred after each addition of base aliquot during titrations to ensure homogeneity and left at a standstill before the fluorescence and absorbance spectra were recorded. Fluorescence titration data were analyzed for the p$K_a$ using the Henderson-Hasselbalch equation from the emission intensity differences, shown by Eq. 1.

$$\mathrm{pH} = \mathrm{p}K_\mathrm{a} + \log\left(\frac{I_{max} - I}{I - I_{min}}\right) \qquad (1)$$

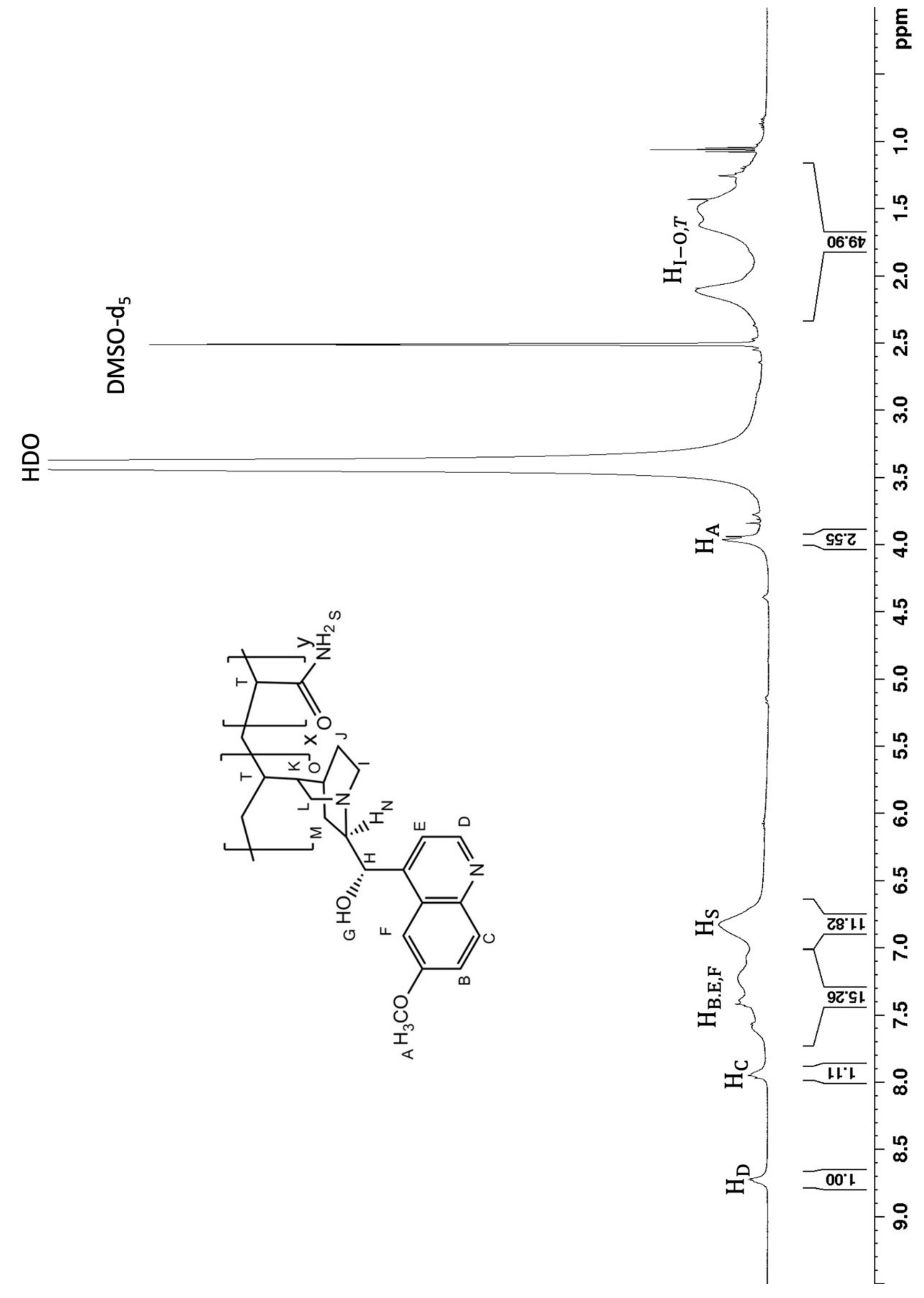

**Fig. S1** $^{1}$H NMR spectrum of **1** in DMSO-$d_6$.

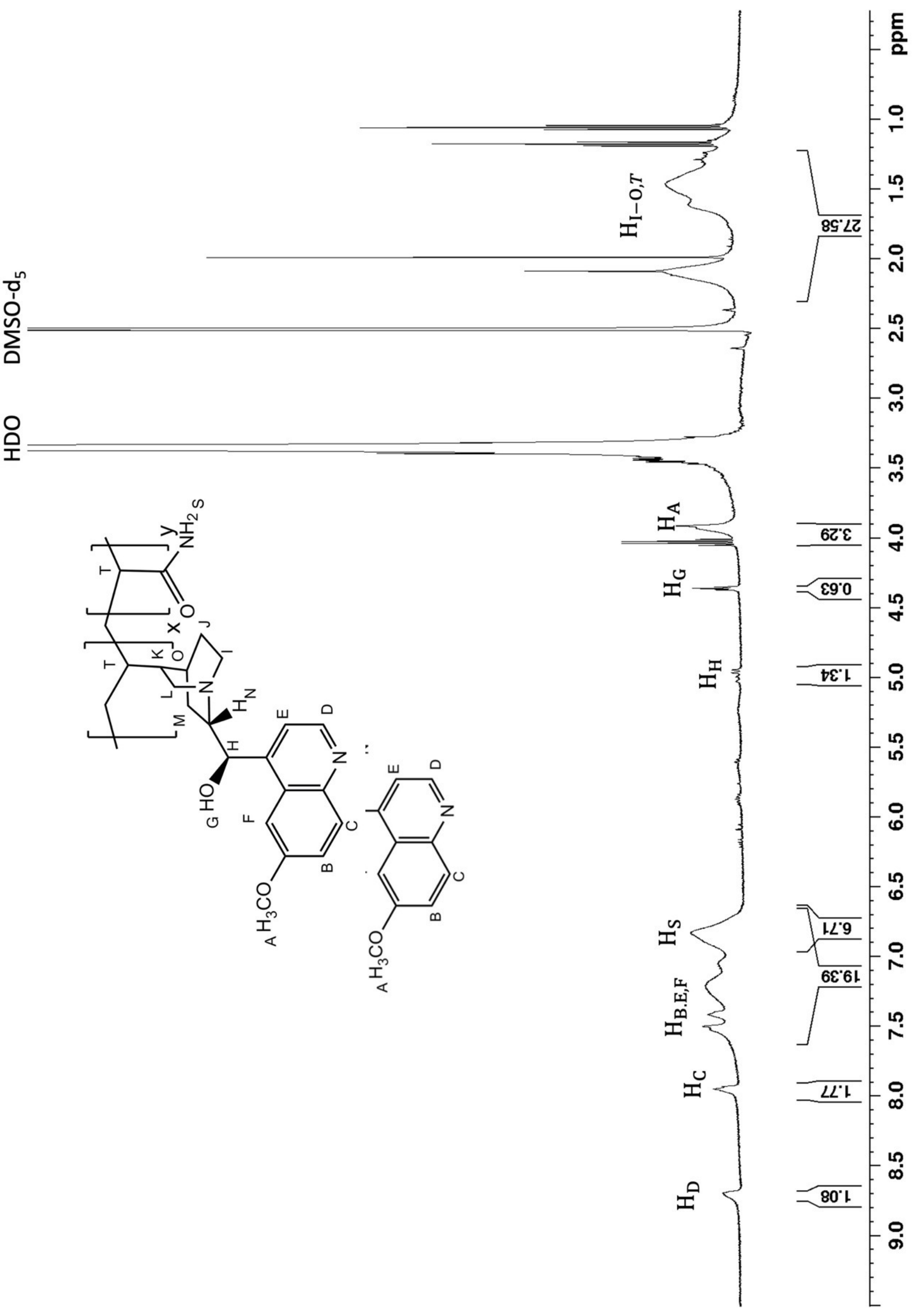


**Fig. S2** $^{1}$H NMR spectrum of **2** in DMSO-$d_6$.

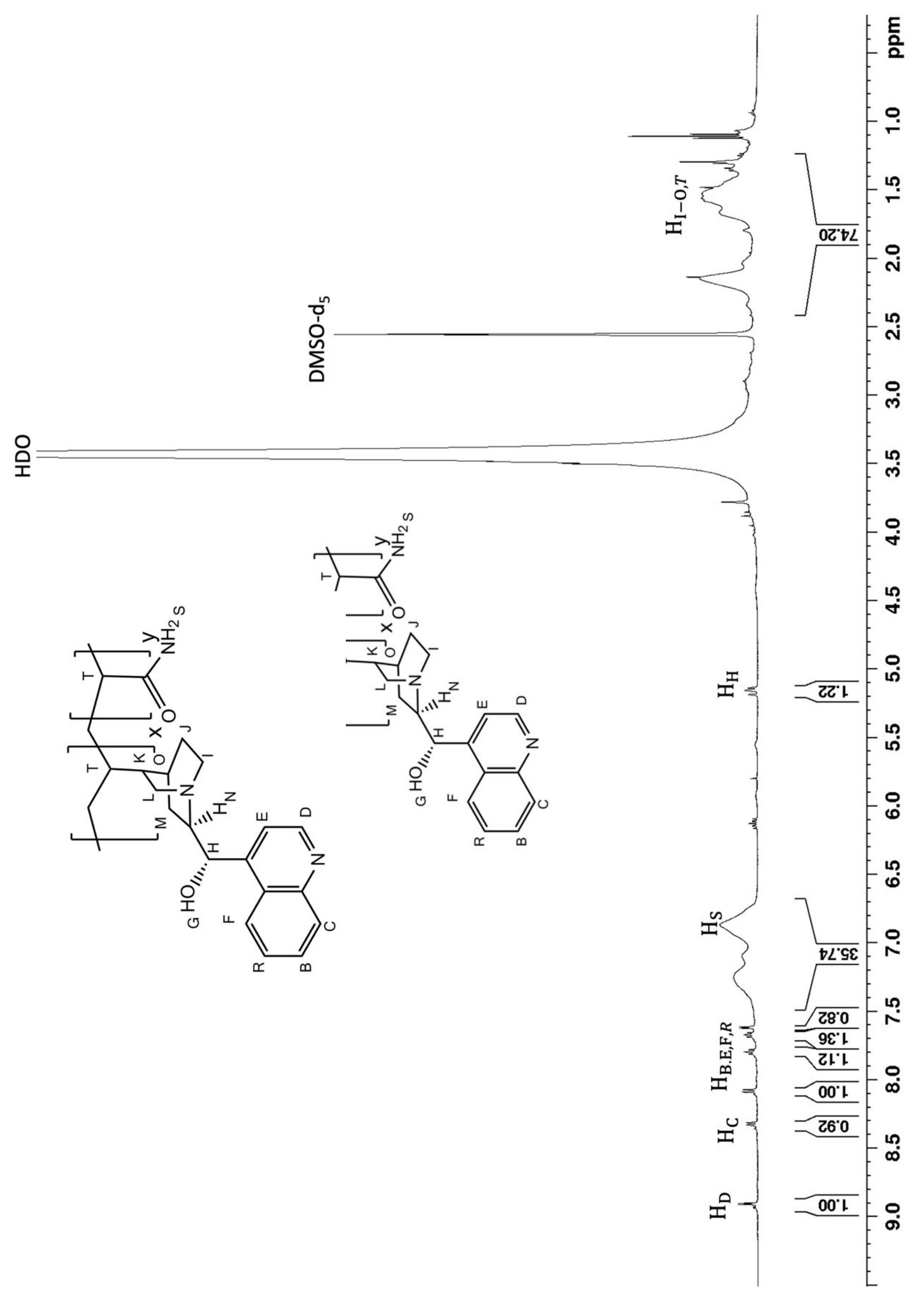


**Fig. S3** $^{1}H$ NMR spectrum of **3** in DMSO-$d_6$.

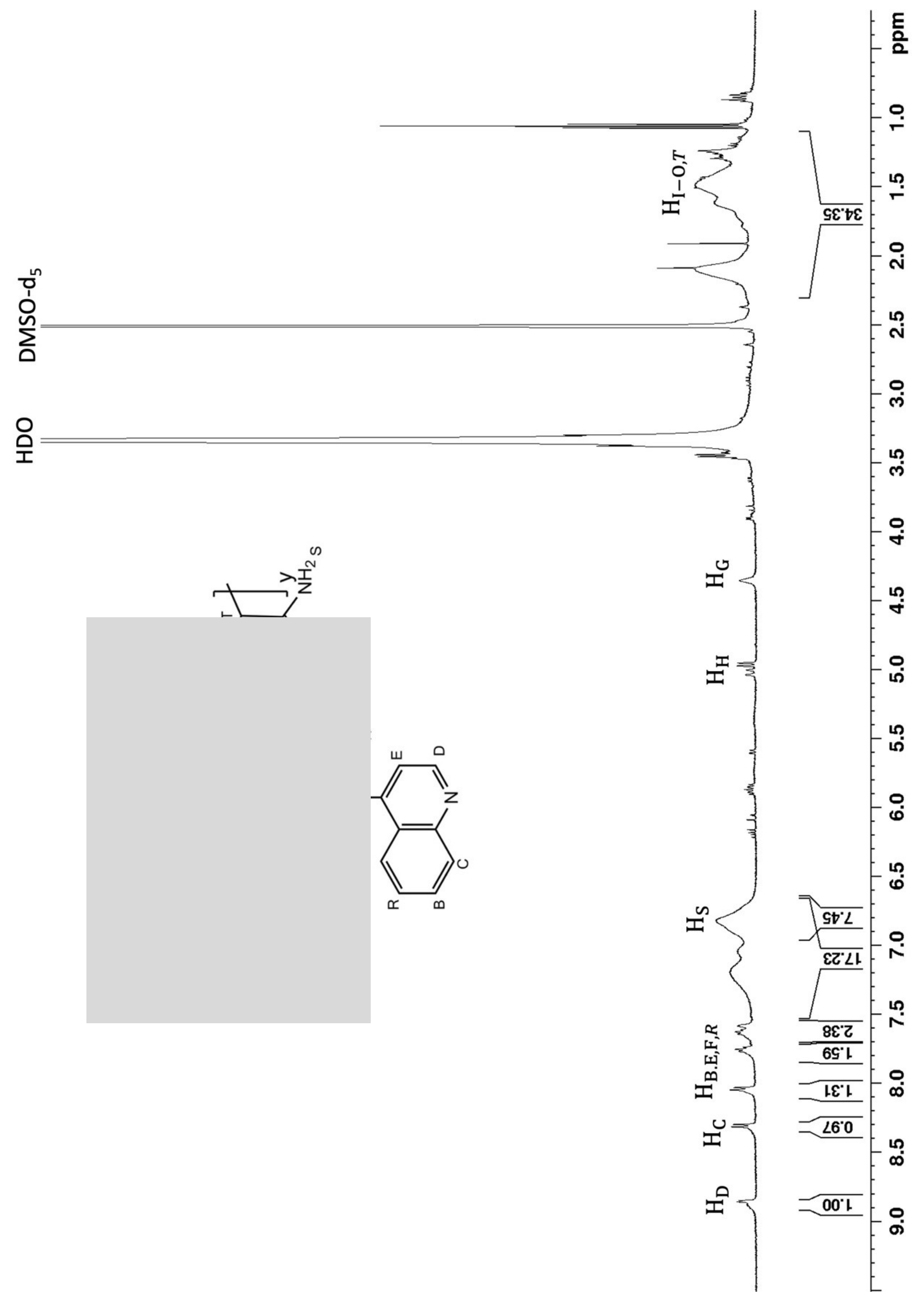


**Fig. S4** $^1$H NMR spectrum of **4** in DMSO-$d_6$.

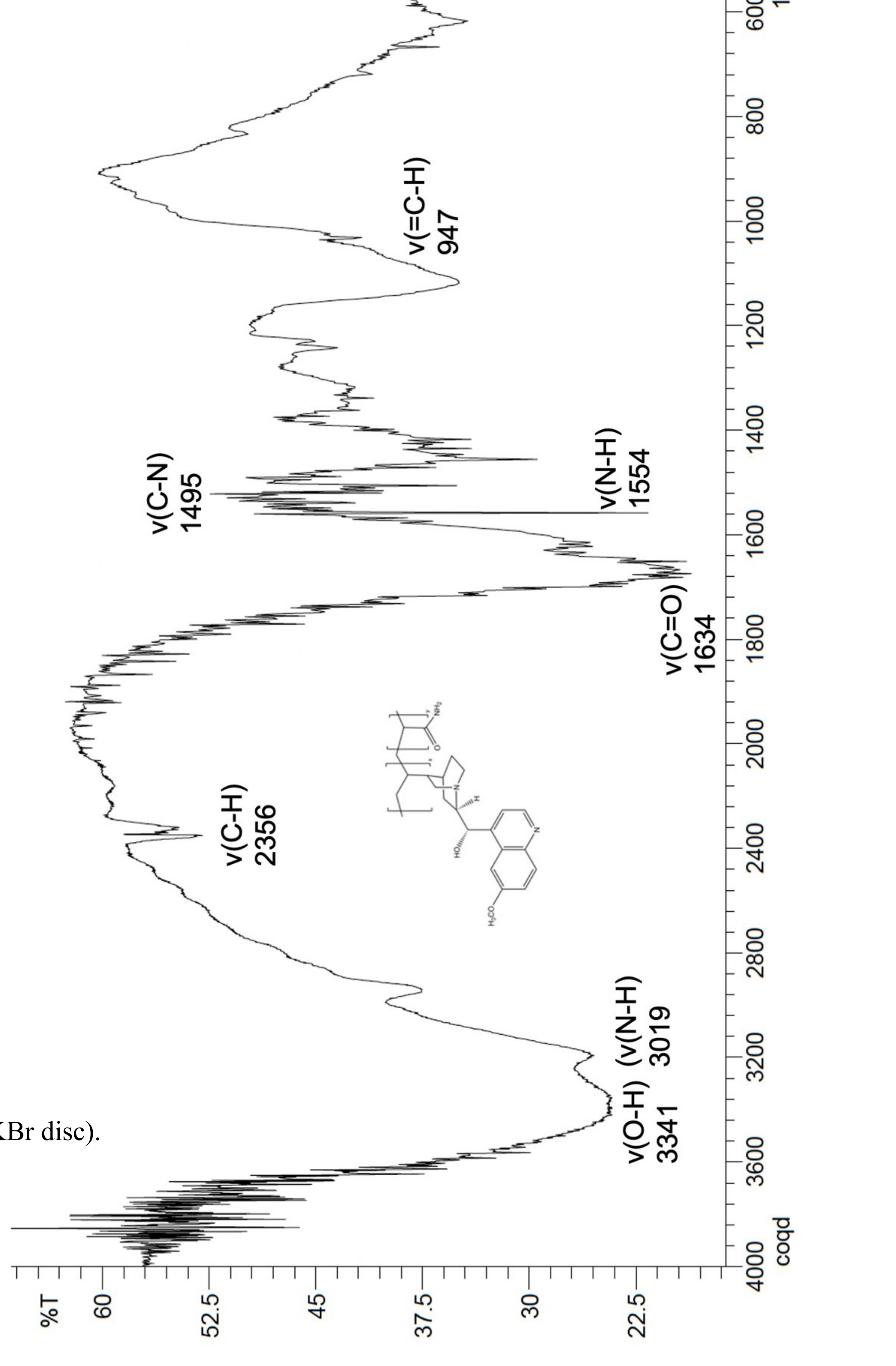


**Fig. S5** IR spectrum of **1** (KBr disc).

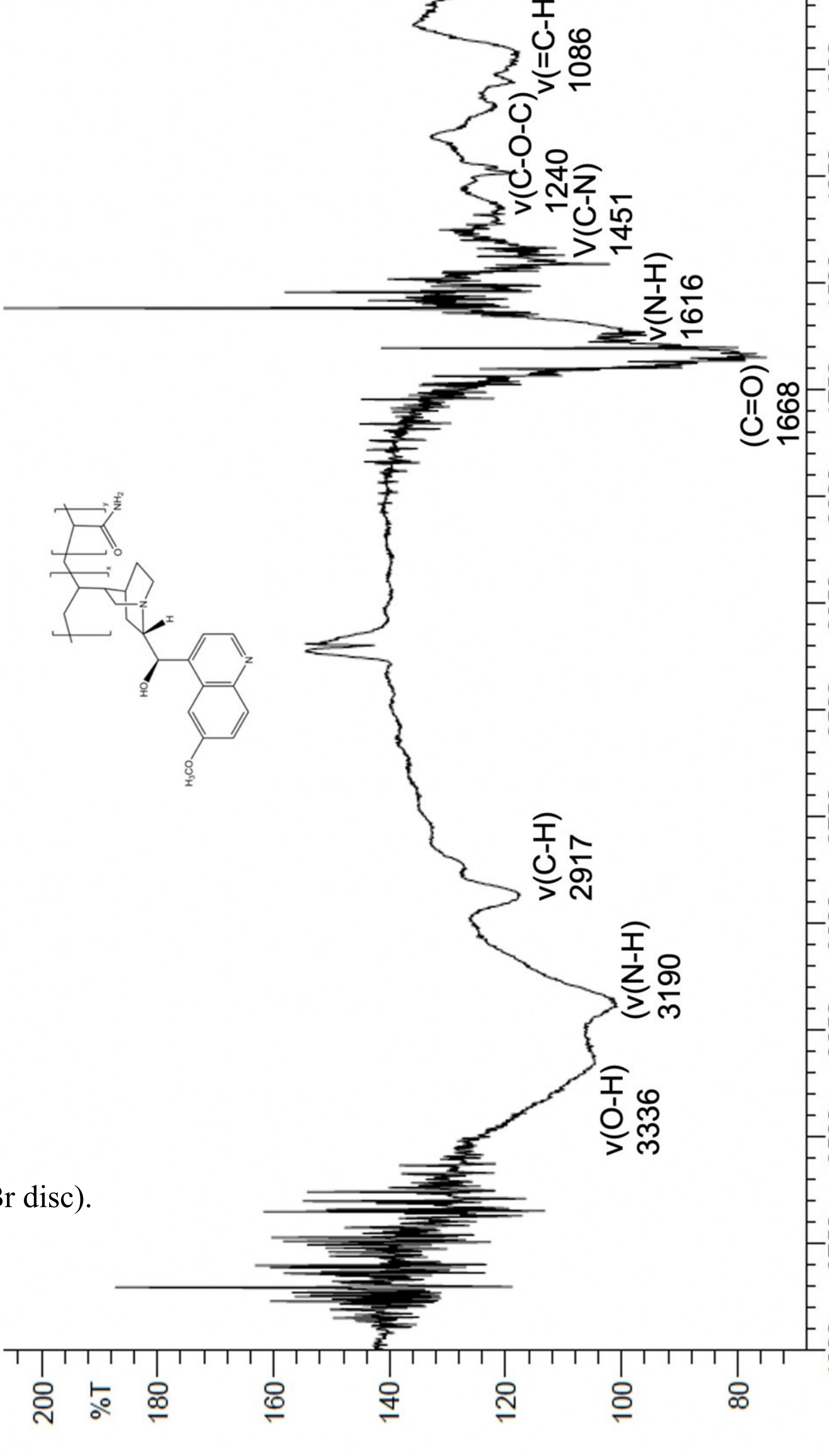


**Fig. S6** IR spectrum of **2** (KBr disc).

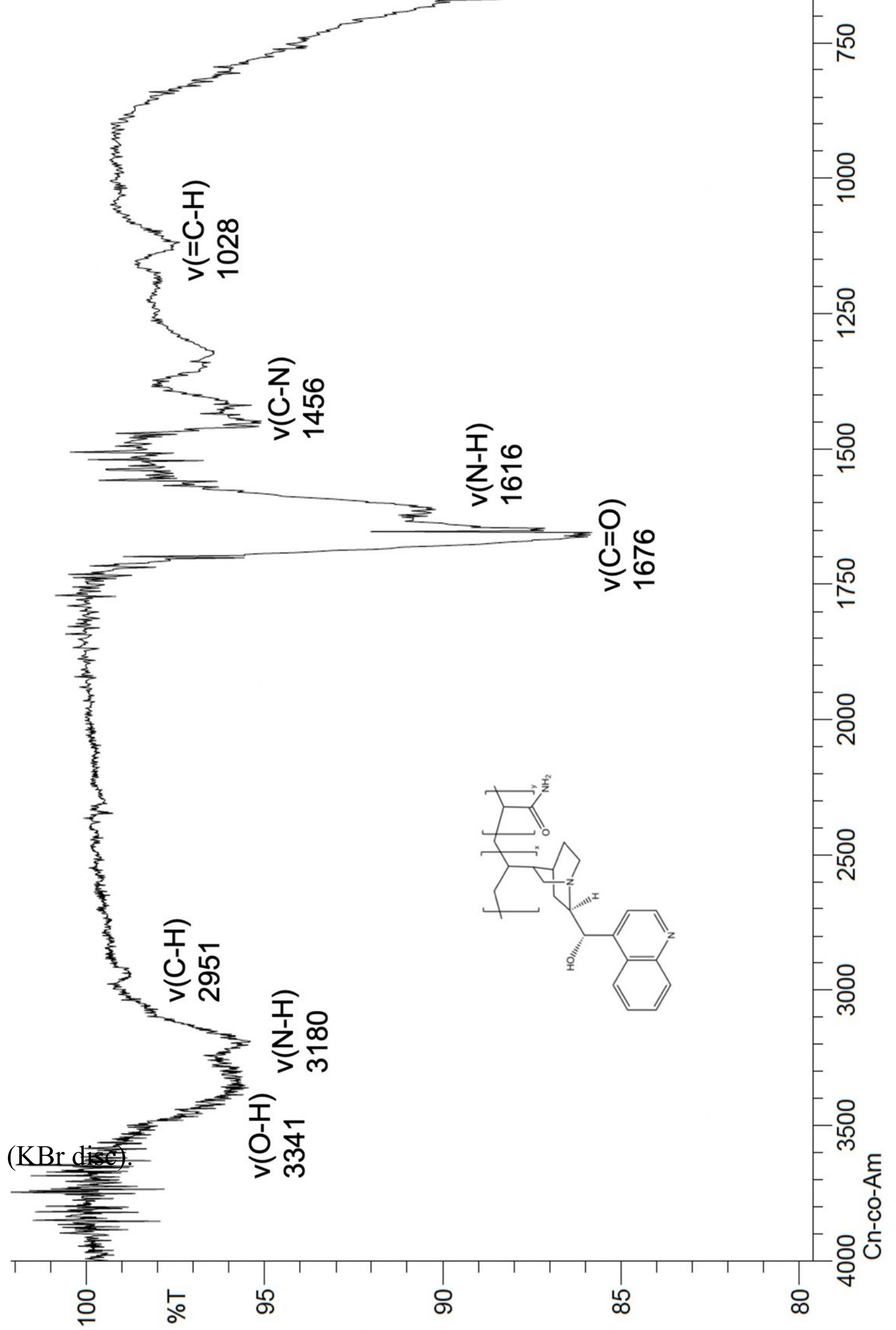


**Fig. S7** IR spectrum of **3** (KBr disc).

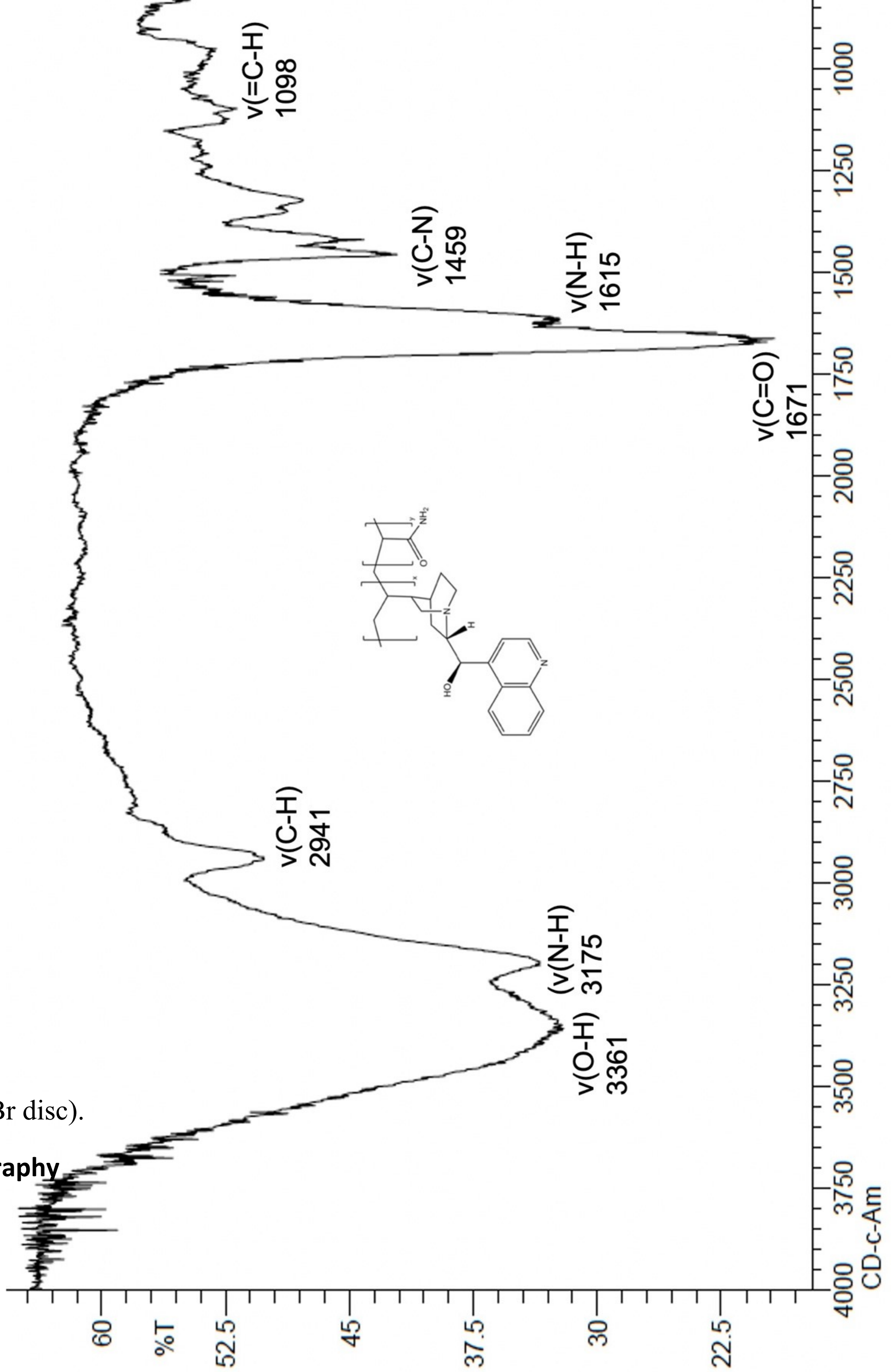


**Fig. S8** IR spectrum of **4** (KBr disc).

**Gel Permeation Chromatography**

**Fig. S9** Gel permeation chromatography of copolymers **1-4** in water to determine the

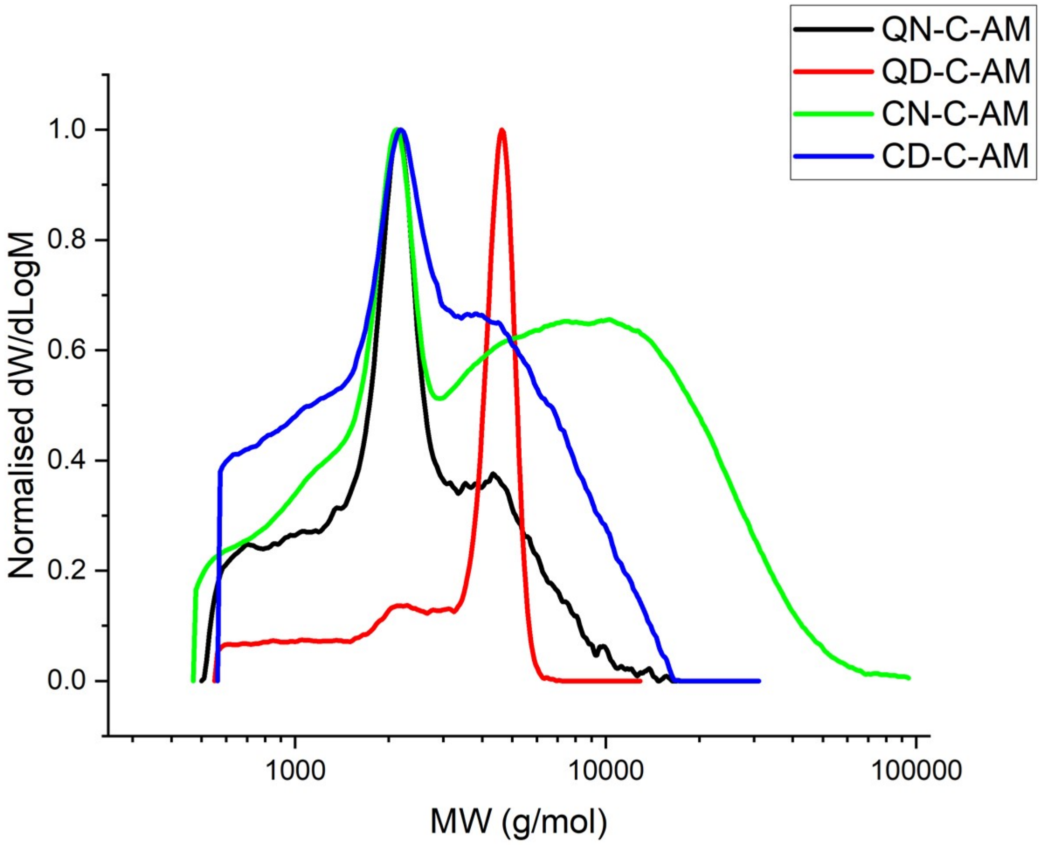


molecular weight distributions: **1** quinidine (QD-C-AM), **2** quinine (QN-C-AM), **3** cinchonine (CN-C-AM) and **4** cinchonidine (CD-C-AM).

**Table S1** The molecular weights ($M_n$ and $M_w$) and polydispersity index (Đ) determined by GPC in water for copolymer **1-4**.[a]

| Sample | $M_n$ (g/mol) | $M_w$ (g/mol) | Đ |
|---|---|---|---|
| **1** (QD-C-AM) | 2400 | 3500 | 1.45 |
| **2** (QN-C-AM) | 1800 | 2700 | 1.56 |
| **3** (CN-C-AM | 2700 | 8500 | 3.10 |
| **4** (CD-C-AM) | 1900 | 3500 | 1.81 |

[a] Size-exclusion chromatography (SEC, GPC) was performed using 2 mg/mL samples with a PL-aquag-OH PL1110-6504 column at a flow rate of 1.0ml/min at 30 °C in water/0.1 M $NaNO_3$ using polyethylene glycol (PEG) as the standard with Mp values between 194-1,250,000. Using the ratios from the NMR data (Table 1) and the GPC data (Table S1) , the mean composition of the copolymers is three *cinchona* alkaloids and 18, 12, 27 and 15 AM repeat units for **1-4**, respectively.

**DLS of copolymer 1 QD-co-AM as a function of concentration and pH**

(a)

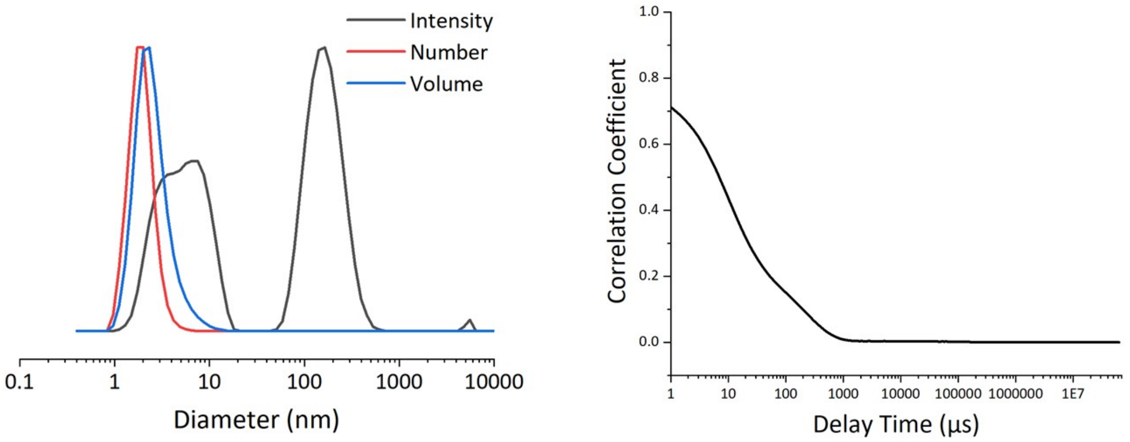


(b)

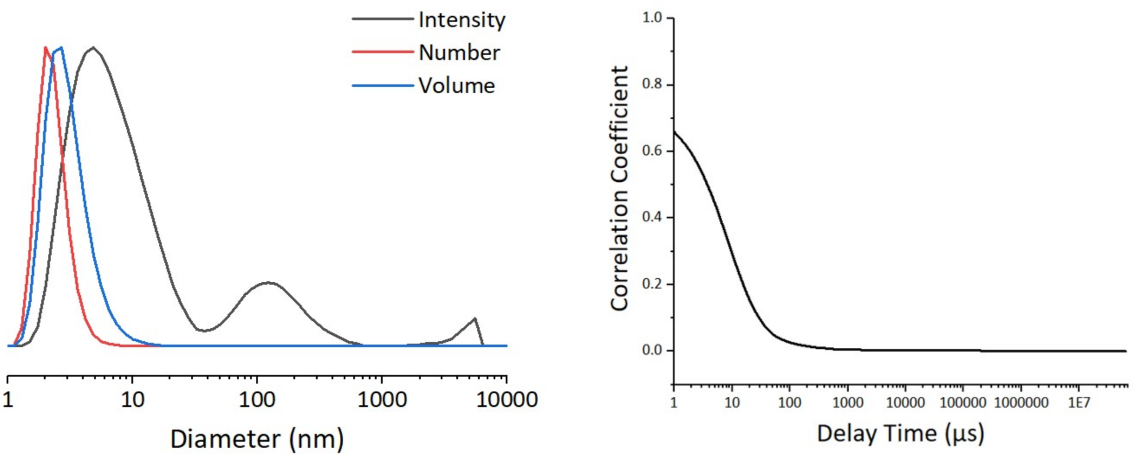


(c)

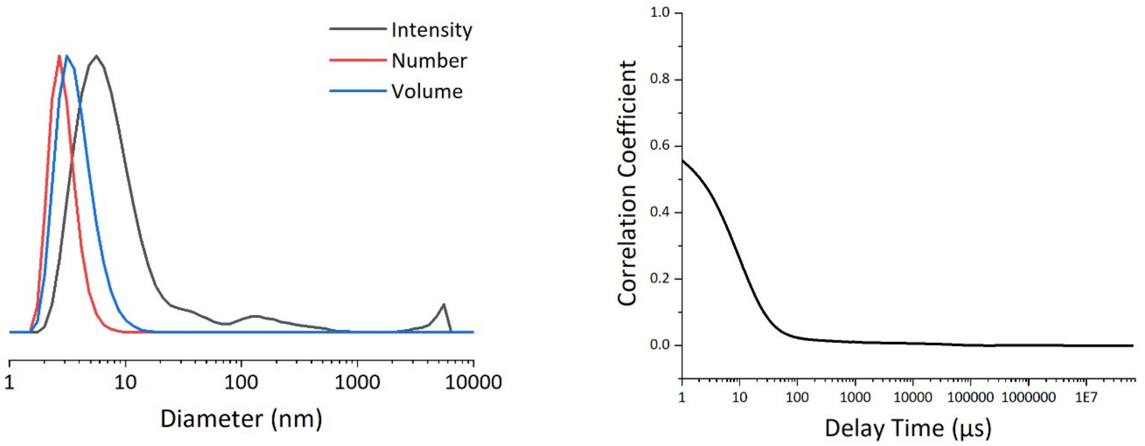


**Fig. S10** DLS trace (above, left) and correlogram (above, right) of copolymer QD-co-AM **1** dissolved in 1 mL deionised water with (a) 5.0 mg at pH 6, (b) 5.0 mg at pH 2 and (c) 2.5 mg at pH 2.

**DLS of copolymer 2 QN-co-AM as a function of concentration at pH 6.**

(a)

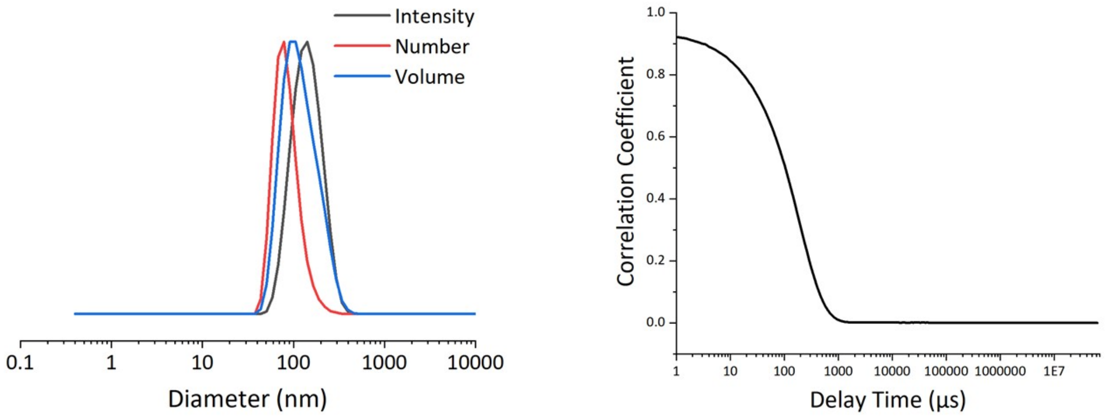


(b)

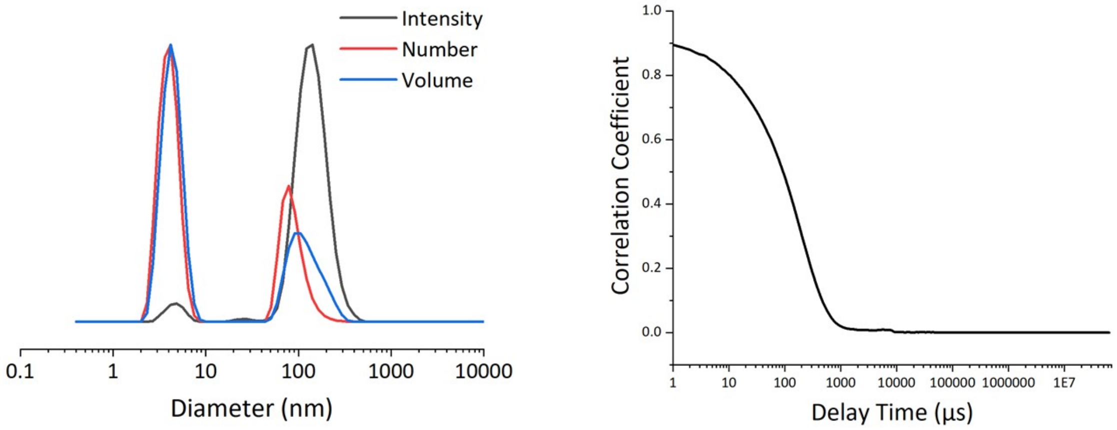


(c)

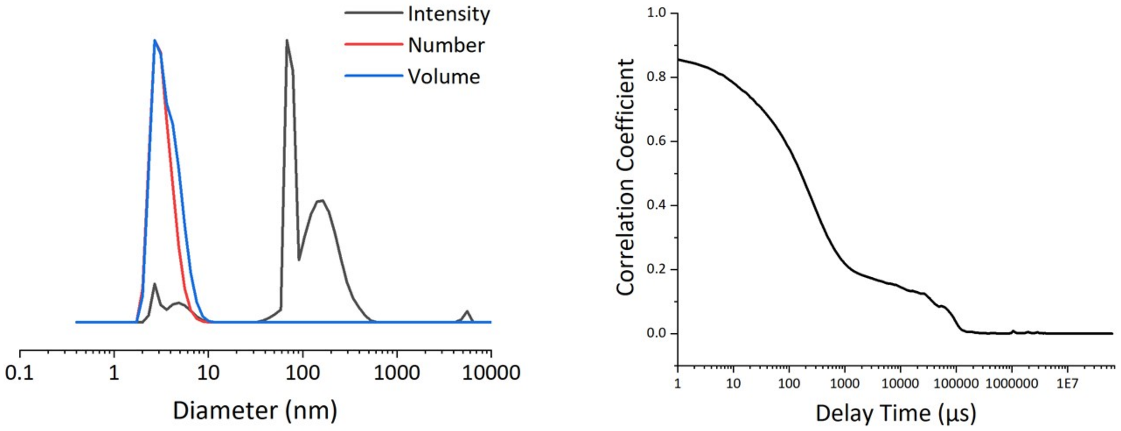


**Fig. S11** DLS trace (above, left) and correlogram (above, right) of copolymer QN-co-AM **2** at pH 6 dissolved in 1 mL deionised water with (a) 5.0 mg, (b) 2.5 mg and (c) 0.5 mg.

**DLS of copolymer 2 QN-co-AM as a function of concentration at pH 2.**

(a)

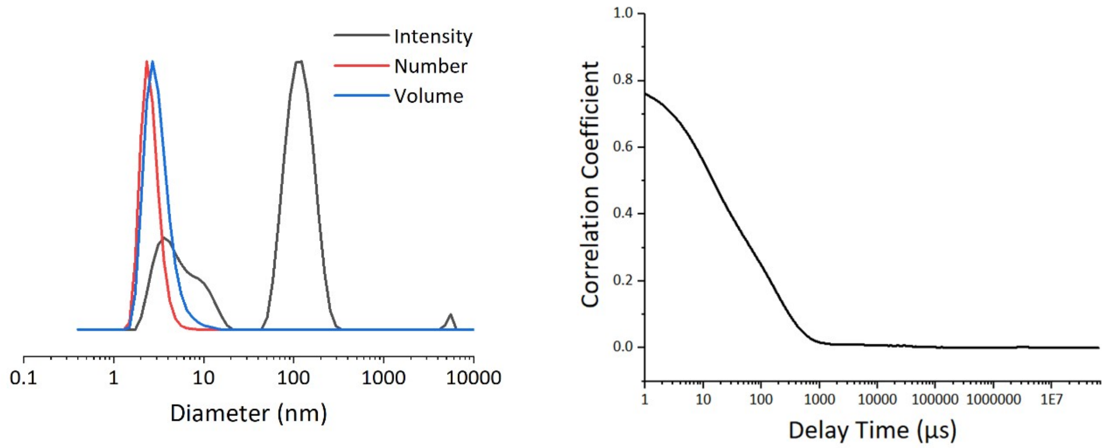


(b)

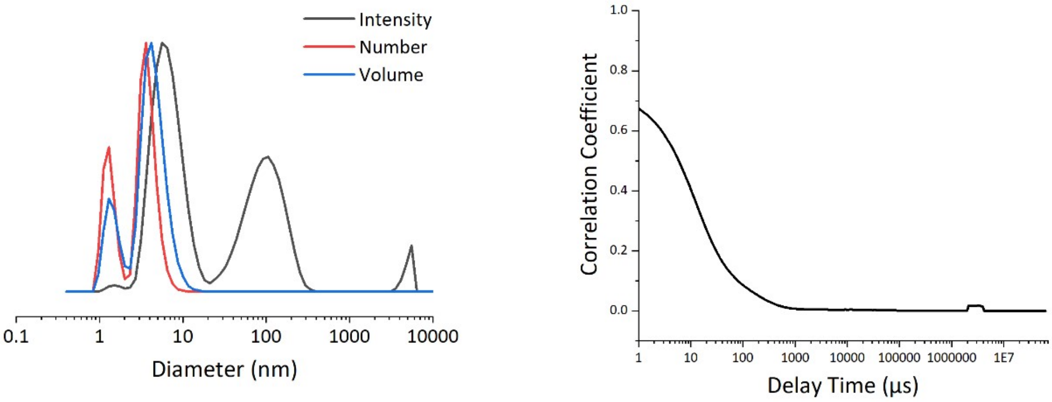


**Fig. S12** DLS trace (above, left) and correlogram (above, right) of copolymer QN-co-AM **2** at pH 2 dissolved in 1 mL deionised water with (a) 5.0 mg and (b) 2.5 mg.

**DLS of copolymer 3 CN-co-AM as a function of concentration and pH.**

(a)

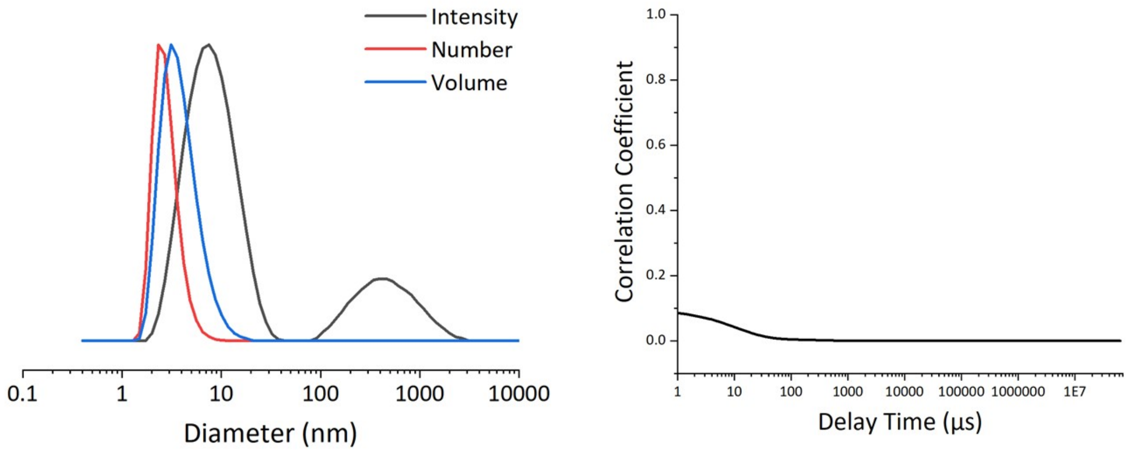


(b)

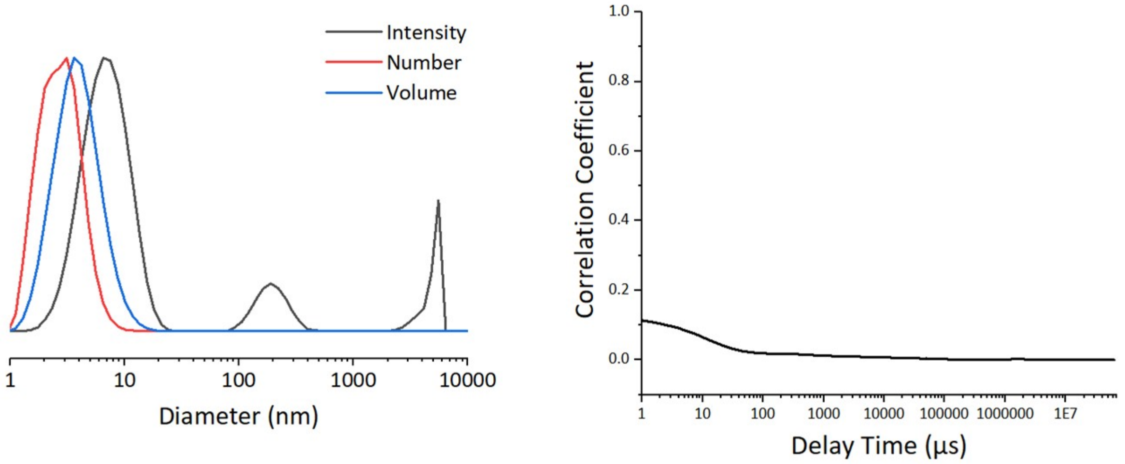


(c)

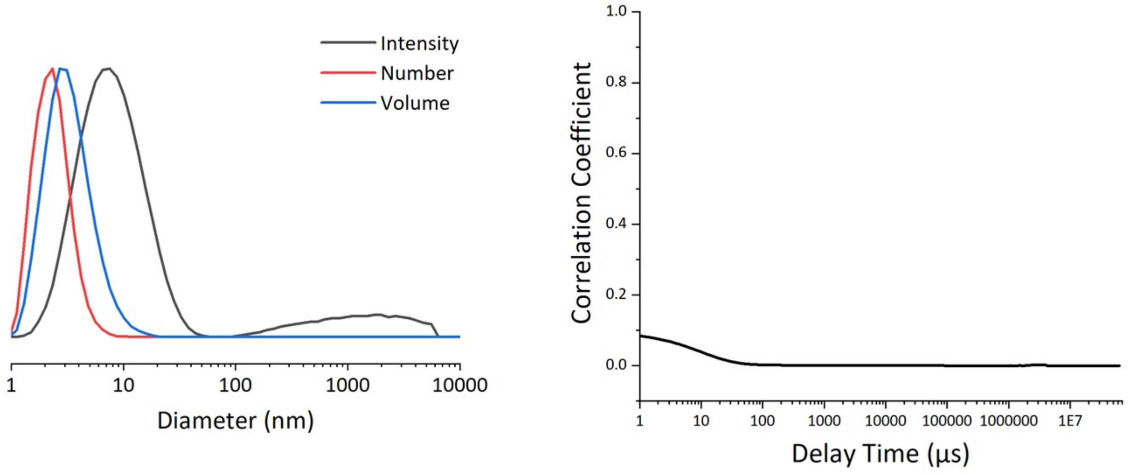


**Fig. S13** DLS trace (above, left) and correlogram (above, right) of copolymer CN-co-AM **3** dissolved in 1 mL deionised water with (a) 5.0 mg at pH 6, (b) 5.0 mg at pH 2 and (c) 2.5 mg at pH 2.

**DLS of copolymer 4 CD-co-AM as a function of concentration and pH.**

(a)

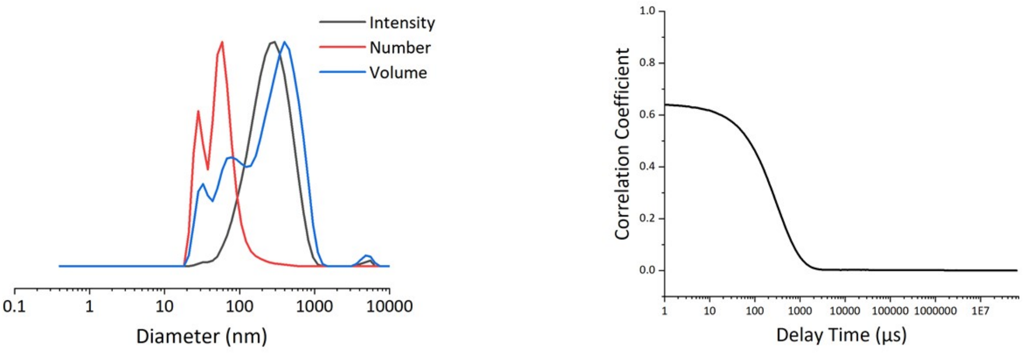


(b)

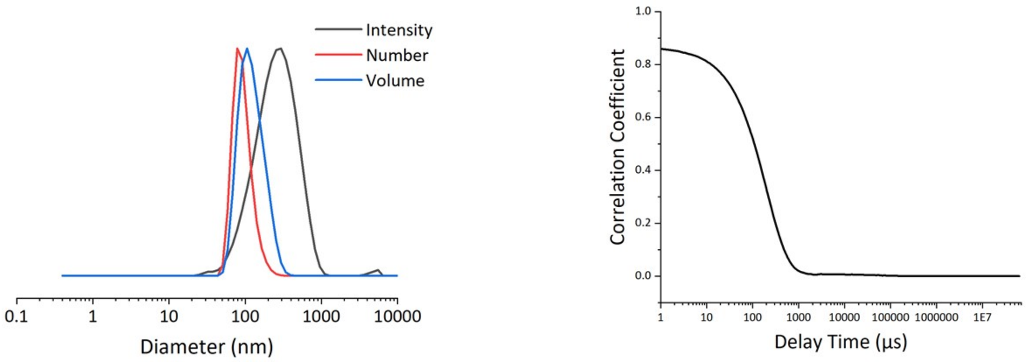


(c)

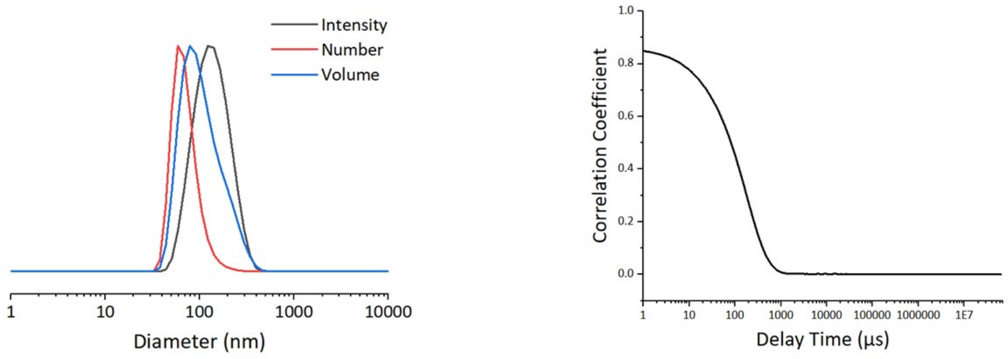


(d)

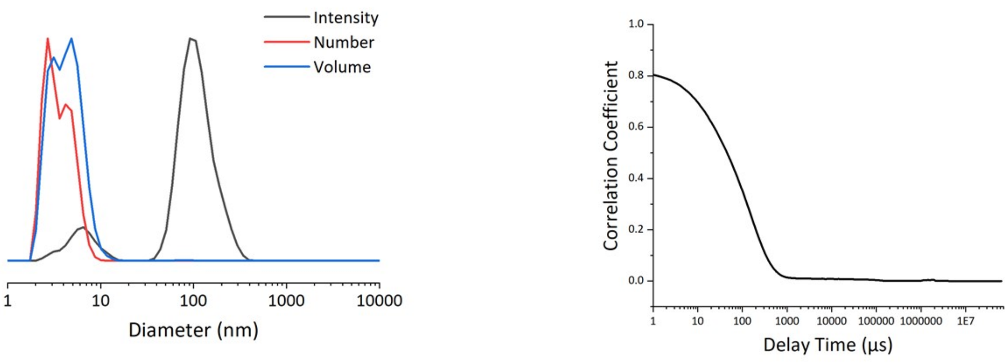


**Fig. S14** DLS trace (above, left) and correlogram (above, right) of copolymer CD-co-AM **4** dissolved in 1 mL deionised water with (a) 5.0 mg at pH 6, (b) 0.5 mg at pH 6, (c) 5.0 mg at pH 2 and (d) 2.5 mg at pH 2.

**UV-vis absorbance spectra of 2 in water**

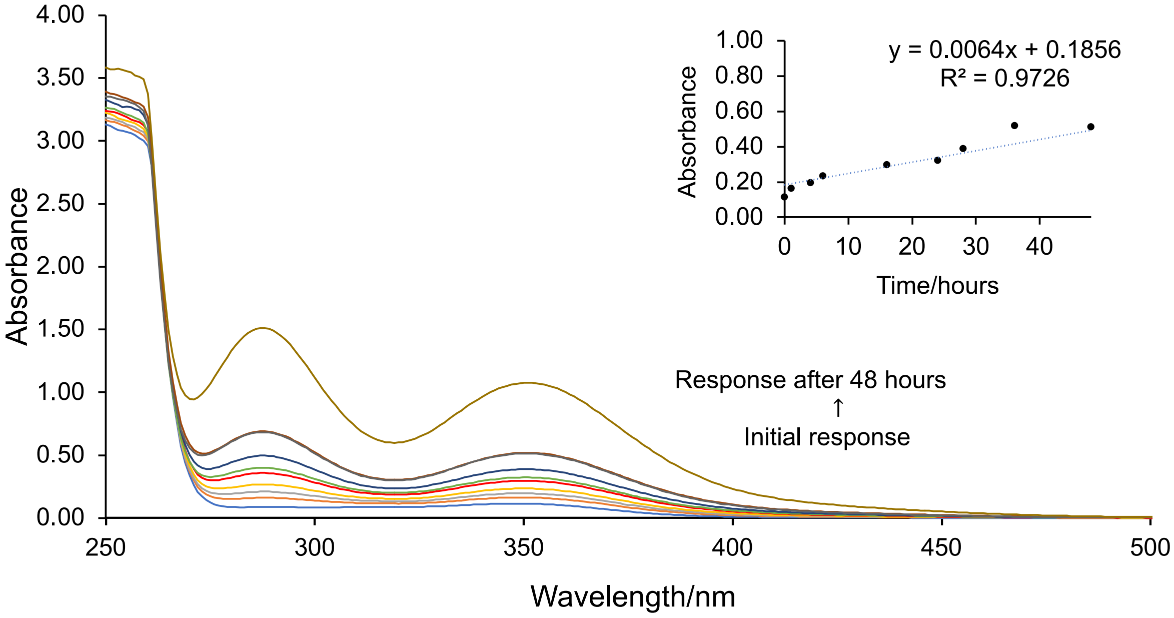


**Fig. S15** UV-vis absorbance spectra of 0.12 g/L **2** in $10^{-2}$ M $H^+$ and 200 mM $I^-$ in water as a function of time. Inset: the absorbance at 350 nm as a function of time.

**UV-vis absorbance spectra of 2 in THF/water**

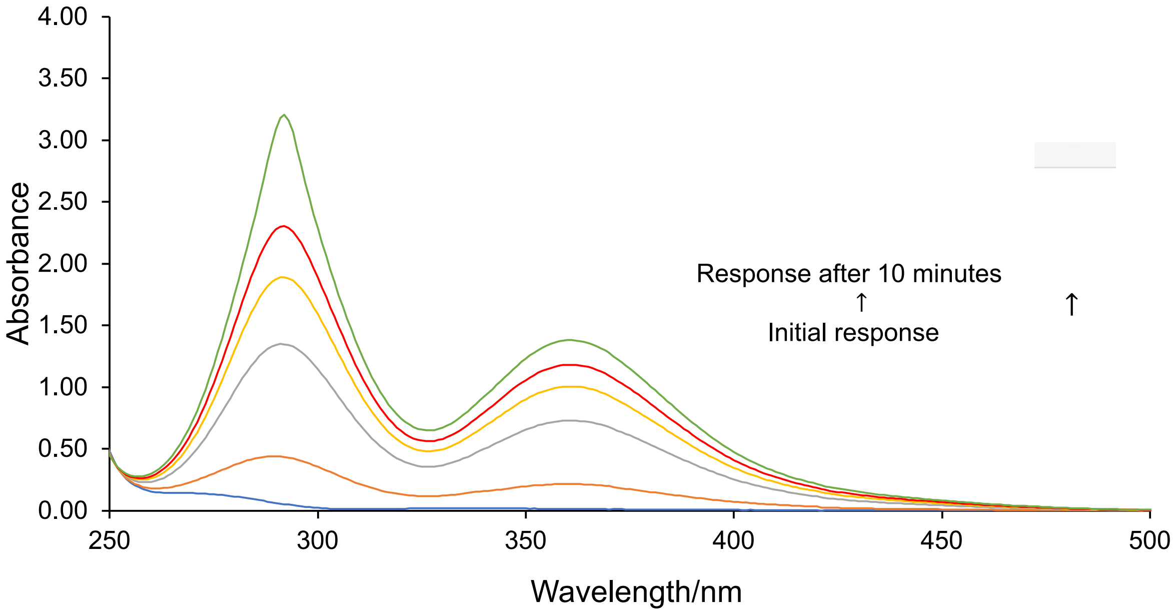


**Fig. S16** UV-vis absorbance spectra of 0.12 g/L of **2** in $10^{-2}$ M $H^+$ and 1 mM $I^-$ in 1:1 (v/v) $THF/H_2O$ as a function of time.

**Vials of 2 demonstrating AND logic in water**

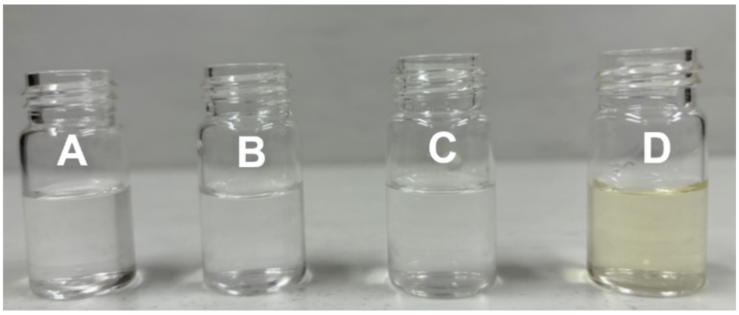


**Fig. S17** Vial containing 0.12 g/L of **2** in water (A) $10^{-9}$ M $H^+$ (B) $10^{-2}$ M $H^+$ (C) $10^{-9}$ M $H^+$ and 200 mM $I^-$ (D) $10^{-2}$ M $H^+$ and 200 mM $I^-$ in water after 48 hours. The copolymer operates as a $H^+$, $I^-$-driven AND logic gate.

**Vials of 2 demonstrating TRANSFER logic in 9:1 (v/v) THF/water**

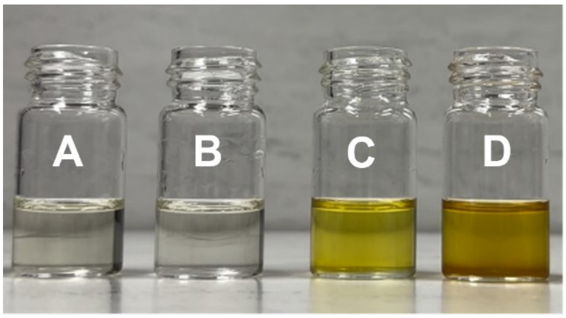


**Fig. S18** Vial containing 0.12 g/L of **2** in 9:1 (v/v) THF/water (A) $10^{-9}$ M $H^+$ (B) $10^{-2}$ M $H^+$ (C) $10^{-9}$ M $H^+$ and 10 μM $I^-$ (D) $10^{-2}$ M $H^+$ and 10 μM $I^-$ after 1 min. The copolymer operates as a $H^+$, $I^-$-driven TRANSFER logic gate.

**UV-vis spectra of 2 demonstrating AND logic in water**

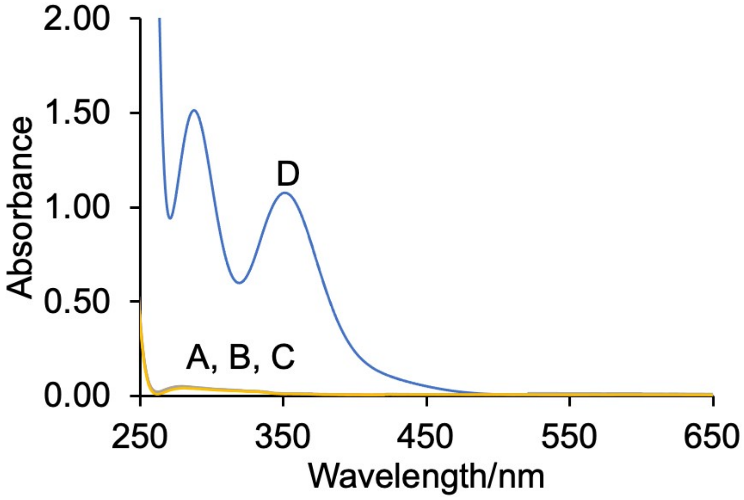


**Fig. S19** UV-vis absorbance spectra of 0.12 g/L of poly(QN-co-Am) **2** in water (A) $10^{-9}$ M $H^+$ (B) $10^{-2}$ M $H^+$ (C) $10^{-9}$ M $H^+$ and 200 mM $I^-$ (D) $10^{-2}$ M $H^+$ and 200 mM $I^-$ after 48 hours. The copolymer operates as a $H^+$, $I^-$-driven AND logic gate with the threshold set at A = 0.50.

**UV-vis spectra of 2 demonstrating TRANSFER logic in 9:1 (v/v) THF/water**

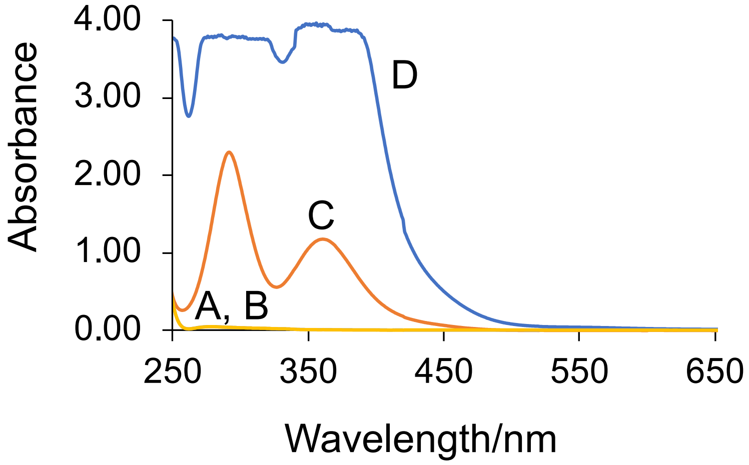


**Fig. S20** UV-vis absorbance spectra of 0.12 g/L of poly(QN-co-Am) **2** in 9:1 (v/v) THF/water (A) $10^{-9}$ M $H^+$ (B) $10^{-2}$ M $H^+$ (C) $10^{-9}$ M $H^+$ and 10 µM $I^-$ (D) $10^{-2}$ M $H^+$ and 10 µM $I^-$ after 1 min. The copolymer operates as a $H^+$, $I^-$-driven TRANSFER logic gate with the threshold set at A = 0.50.

**Table S2**. The polydispersity index (PDI) values for copolymers **1-4**.

| Polymer | Concentration (mg/mL) | pH | PDI[a] | Diameter/nm[b] Mean | SD |
|---|---|---|---|---|---|
| QD-co-Am **1** | 5.0 | 6.0 | 0.694 | 1.97 | 0.62 |
| | 5.0 | 2.0 | 0.340 | 2.35 | 0.71 |
| | 2.5 | 2.0 | 0.186 | 3.00 | 0.88 |
| QN-co-Am **2** | 5.0 | 6.0 | 0.269 | 87.02 | 30.78 |
| | 2.5 | 6.0 | 0.434 | 32.36 | 43.47 |
| | 0.5 | 6.0 | 0.477 | 3.32 | 0.92 |
| | 5.0 | 2.0 | 0.840 | 2.60 | 0.67 |
| | 2.5 | 2.0 | 0.539 | 3.05 | 1.45 |
| CN-co-Am **3** | 5.0 | 6.0 | 0.377 | 2.86 | 0.92 |
| | 5.0 | 2.0 | 0.255 | 2.92 | 1.25 |
| | 2.5 | 2.0 | 0.220 | 2.43 | 0.94 |
| CD-co-Am **4** | 5.0 | 6.0 | 0.269 | 57.78 | 37.52 |
| | 0.5 | 6.0 | 0.216 | 94.40 | 29.34 |
| | 5.0 | 2.0 | 0.289 | 72.32 | 25.70 |
| | 2.5 | 2.0 | 0.451 | 3.71 | 1.29 |

[a] PDI values calculated using PDI = $(\sigma/d)^2$ whereby $\sigma$ is the standard deviation (SD) and $d$ is the mean diameter.[S1] [b] Determined by DLS from the red curve (Number) in Fig. S10-S14.

**Reference**:

S1. N. Raval, R. Maheshwari, D. Kalyane, S. R. Youngren-Ortiz, M. B. Chougule, R. K. Tekade, in Advances in Pharmaceutical Product Development and Research, Basic Fundamentals of Drug Delivery, Academic Press, 2019, Chapter 10, p. 373-374.
